# Common Altered Epigenomic Domains in Cancer Cells: Characterization and Subtle Variations

**DOI:** 10.3390/cancers3021996

**Published:** 2011-04-18

**Authors:** Yi-Chien Tsai, Chun-Hui Chiao, Ian Yi-Feng Chang, Dow-Tien Chen, Tze-Tze Liu, Kate Hua, Chuan-Hsiung Chang, Ming-Ta Hsu

**Affiliations:** 1 Institute of Molecular Biology and Biochemistry, National Yang Ming University, No.155, Sec.2, Linong Street, Taipei, 112 Taiwan; E-Mails: eva@prismabiotech.com.tw (Y.C.T.); anna19831205@yahoo.com.tw (C.H.C.); 2 VGH-YM Genome Center, National Yang Ming University, No.155, Sec.2, Linong Street, Taipei, 112 Taiwan; E-Mails: dtchen@ms6.rul.com.tw (D.T.C.); ttliu@ym.edu.tw (T.T.L.); katherine_hua@hotmail.com (K.H.); 3 Institute of Biomedical Informatics, National Yang Ming University, No.155, Sec.2, Linong Street, Taipei, 112 Taiwan; E-Mails: cif077@yahoo.com.tw (I.Y.F.C.); cchang@ym.edu.tw (C.H.C.)

**Keywords:** epigenetic, DNA methylation, histone modification, cancer

## Abstract

We have previously identified large megabase-sized hypomethylated zones in the genome of the breast cancer cell line MCF-7 using the TspRI-ExoIII technique. In this report, we used a more convenient high throughput method for mapping the hypomethylated zones in a number of human tumor genomes simultaneously. The method was validated by the bisulfite sequencing of 39 randomly chosen sites in a demethylated domain and by bisulfite genome-wide sequencing of the MCF-7 genome. This showed that the genomes of the various tumor cell lines, as well as some primary tumors, exhibit common hypomethylated domains. Interestingly, these hypomethylated domains are correlated with low CpG density distribution genome-wide, together with the histone H3K27Me3 landscape. Furthermore, they are inversely correlated with the H3K9Ac landscape and gene expression as measured in MCF-7 cells. Treatment with drugs resulted in *en-bloc* changes to the methylation domains. A close examination of the methylation domains found differences between non-invasive and invasive tumors with respect to tumorigenesis related genes. Taken together these results suggest that the human genome is organized in epigenomic domains that contain various different types of genes and imply that there are cis- and trans-regulators that control these domain-wide epigenetic changes and hence gene expression in the human genome. The hypomethylated domains are located in gene deserts that contain mainly tissue-specific genes and therefore we hypothesize that tumor cells keep these regions demethylated and silenced in order to save energy and resources and allow higher levels of cell proliferation and better survival (a thrifty tumor genome hypothesis).

## 1. Introduction

DNA methylation and histone modifications constitute the major epigenetic mechanisms involved in regulating mammalian gene expression [1-3]. Although methylation of the promoters of genes has been shown to be associated with gene silencing [4,5], there is little information available on the role of intragenic methylation, or on genome-wide methylation, in terms of overall gene expression regulation. Recently, a number of methods have been developed that allow the overall CpG methylation present in the genome to be determined. These include affinity enrichment of methylated sequences [6-9], methylation-sensitive restriction endonuclease digestion followed by amplification of the resistant DNA and direct bisulfite sequencing using a high throughput sequencing platform [10]. Although direct bisulfite sequencing can produce results with single nucleotide resolution, the method is very expensive and requires expertise in bioinformatics in order to map the bisulfite converted sequences back to the genome and some highly converted short sequences are difficult to map. The mC antibody or methylation binding proteins needed in the affinity enrichment methods are not readily available. To provide a high throughput method for mapping overall methylation pattern in a genome, we previously developed a biochemical method to remove sequences that contain unmethylated HpaII sites [1]. Using this method we have shown that there are megabase-sized hypomethylated domains in breast tumor cell lines. In this report, we further improve this method using the McrBC enzyme to enrich for hypomethylated large DNA fragments. This high throughput method allows us to examine the methylation pattern of many genomes simultaneously. The results show that genomes from several different tumor types all exhibit similar methylome patterns, albeit with subtle variations. This methylome domain pattern was found to be correlated with various histone modification landscapes, with CpG density distribution, gene density and gene expression. We suggest that this implies that tumor cells keep these regions demethylated and silenced in order to save energy and resources. This would allow higher levels of cell proliferation and better survival of the tumor cells and supports a thrifty tumor genome hypothesis.

## 2. Results

### 2.1. Rapid Mapping of Demethylated Domains in Tumor Genomes by McrBC-Array

**Figure 1.**
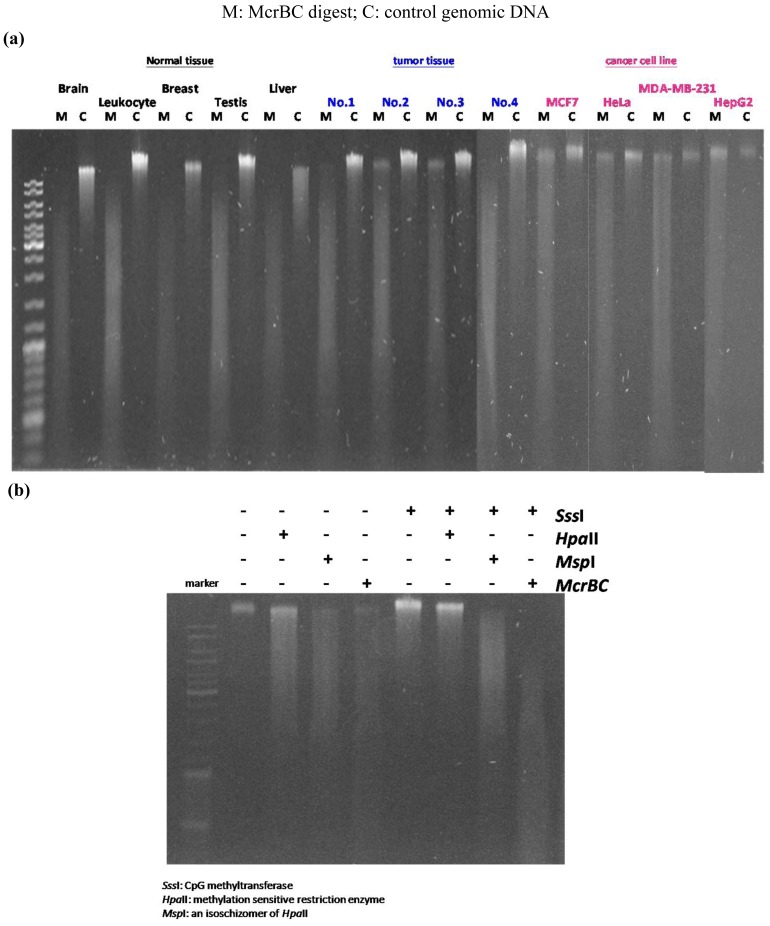
Genomic DNA digested by McrBC. (**a**) Digestion of normal tissue and tumor DNA with McrBC enzyme. Tumors DNA from primary breast tumors and from various tumor cell lines show the presence of McrBC-resistant high molecular weight DNA, whereas the DNAs from normal tissues are cleaved into small fragments. (**b**) The large pieces of McrBC-resistant MCF-7 DNA became sensitive to McrBC digestion after *in vitro* CpG methylation by SssI methylase (compare 4th lane with the 8th lane).

**Figure 2.**
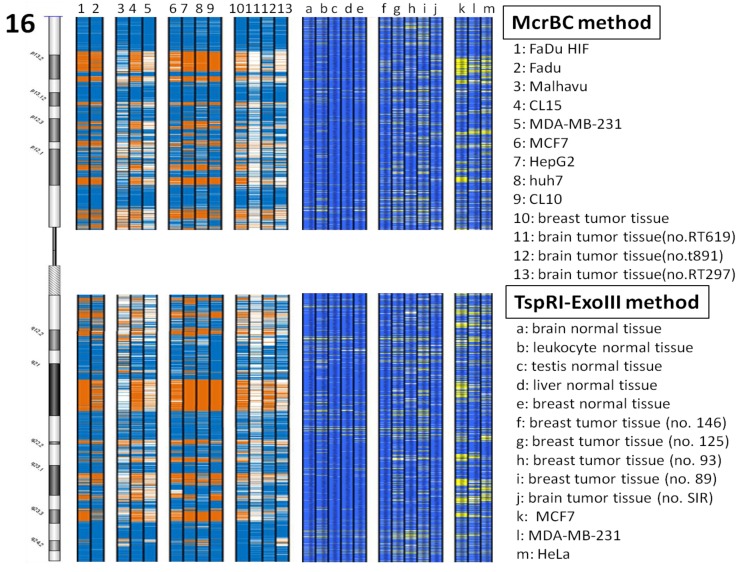
Domain-organization of CG methylation in tumor genomes using chromosome 16 as an example. Lanes 1-13, methylation pattern of tumor genomes analyzed by McrBC-array. The left figure represents chromosome 16, lanes 1-9 represents the methylation patterns of the tumor cell lines for this chromosome; lanes 10-13 represent primary tumors derived from the breast and brain. Lanes a-h, methylation pattern obtained by TspRI-ExoIII method. Lanes a-e are from adult brain, leukocytes, testis, liver and breast respectively. Lanes g-h represents the methylation pattern of MCF-7, MDA-MB-231 and HeLa, respectively. Yellow and orange represent the hypomethylation regions; while blue represent the hypermethylation regions.

In our previous study we developed the TspRI-ExoIII method that allows the rapid mapping of methylated regions in the human genome [1]. Using this method we discovered the presence of large megabase-sized hypomethylated domains in a breast tumor genome. Since this technique requires several biochemical steps before the arrayCGH analysis, we have since developed a more rapid method that allows an even faster analysis of methylome patterns in multiple genomes. We reason that hypomethylated regions should be resistant to the McrBC restriction enzyme, which cleaves methylated DNA [12]. Digestion of tumor DNA with McrBC indeed showed the presence of high molecular weight resistant DNA at the top of the gel. In contrast, no large resistant DNA was found using DNA from the normal human genome (Figure 1a). Furthermore, when tumor DNA was methylated *in vitro* with SssI enzyme before digestion, the McrBC-resistant large fragments were converted into small fragments, indicating that they are resistant to McrBC as a result of hypomethylation (Figure 1b). We characterized the high molecular weight McrBC-resistant DNA near the gel top by elution from the gel and analyzed the fragments using the arrayCGH approach with DNA not digested with McrBC as control (Figure 2). The ratio of signal (McrBC-undigested DNA signal/total genomic DNA signal) >1 is considered to be hypomethylation, while a fold change <1 might be considered to be hypermethylation. However, in order to be more stringent, we defined a fold change of >2 as hypomethylation and these are indicated in orange in Figure 2. On the other hand, when the fold change was <0.5, this was defined as hypermethylation and is indicated in blue. Using this approach, we were able to map the methylation pattern of various tumor genomes. As shown in Figure 2, the patterns of resistant DNA matched those of the hypomethylated zones mapped by the TspRI-ExoIII method in MCF-7 genome and confirmed the presence of large hypomethylated zones in the MCF-7 genome. We confirmed the methylation pattern by bisulfite sequencing of 39 randomly chosen sites from the hypomethylated domain in chromosome 16 that contains the large 1.69 Mb A2BP1 gene using the MCF-7 genome. All 39 sites, including 18 Alu sequences each of about 500 bp, were found to be fully demethylated (data in supplementary). Furthermore, we also confirmed the hypomethylated domain patterns by direct bisulfite sequencing of MCF-7 genome using a Solexa high throughput sequencing method (unpublished data) [13].

### 2.2. The Common Large Hypomethylated Domains Present in Tumor Genomes are Correlated with the CpG Density Distribution and with Gene Density

**Figure 3.**
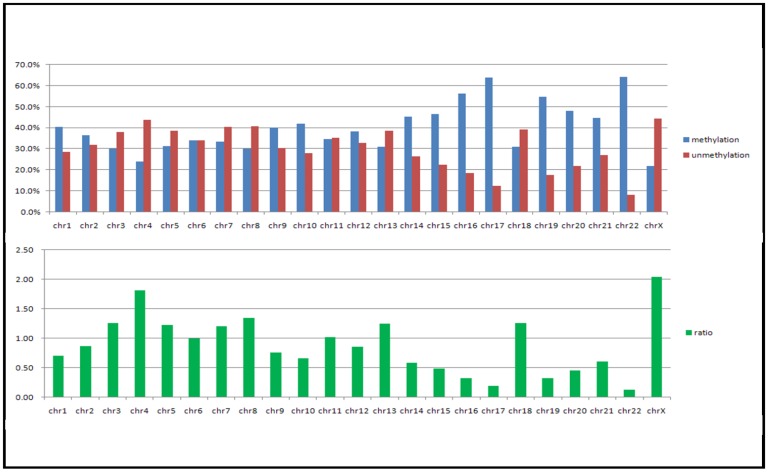
Degree of hypomethylation across the various chromosomes of the MCF-7 genome. The relative number of probes in the array that are hypermethylated (blue) and hypomethylated (red) are plotted for each chromosome (Upper graph). The ratio of the hypomethylated probes to the hypermethylated probes are shown in the lower graph. The X chromosome and chromosome 4 are the most hypomethylated chromosomes, whereas chromosomes 7 and 22 are the most hypermethylated chromosomes.

**Figure 4.**
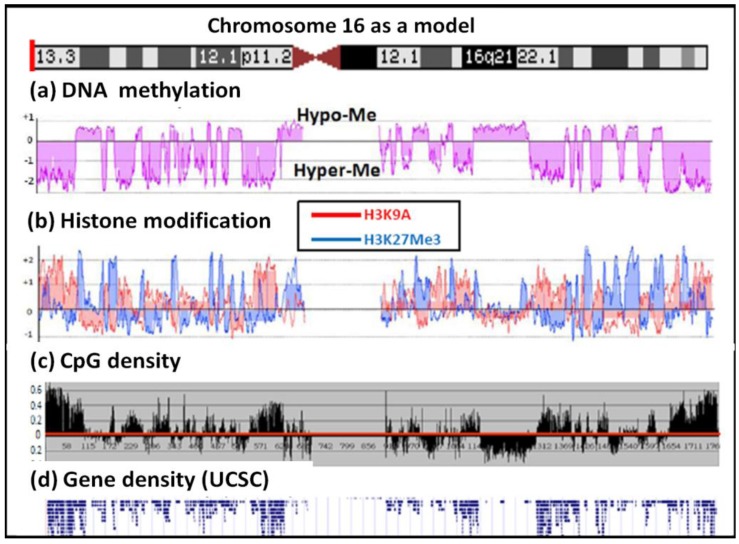
Domain organization in chromosome 16: (a) CG methylation; (b) histone H3K9Ac/H3K27Me3 modification; (c) CpG density and (d) gene density. The CpG density is calculated as the logarithm of the number of CpG sequences within a 50 kb window and the log value is increased by two to exhibit the differential density pattern.

Using the new McrBC method we determined the methylome profiles of 13 human tumor genomes; these included breast, liver, brain and lung tumor cell lines as well as various primary tumor tissues. As shown in Figure 2 and in the supplementary data, all the tumor genomes, including the primary tumors, contained similar hypomethylated domains. As shown in Figure 3, chromosome 22 is the most hypermethylated of all the human chromosomes, whereas the X chromosome and chromosome 4 are the most hypomethylated. It is interesting to note that the common tumor methylome pattern across the various genomes has an interesting one-to-one correspondence with the CpG dinucleotide distribution density in the genome and with gene density (Figure 4).

### 2.3. Subtle Changes in DNA Methylation between Invasive and Non-invasive Cell Lines

Although the gross methylome pattern of tumor genomes looks similar, close examination of methylation patterns between the invasive (HeLa, MDA-MB-231 and CL1-5) and non-invasive cell lines (MCF-7, CL1-0) showed subtle differences in the methylation of genes between these cell lines. For example, there are 404 hypermethylated sites in invasive cell line genomes but are hypomethylated in MCF-7 and normal tissue genomes. These differentially methylated sites are mainly located in large genes (>100 kb). For example, the large fragile site genes DAB1, ESRRG, USH2A are hypomethylated in MCF-7 but hypermethylated in the invasive cell lines. A number of these sites were validated by bisulfite sequencing. Among the genes differentially methylated are number of well- known tumor suppressor genes, namely RASSF1, DLG1, DLC1 and PRDM2, as well as various other tumor related genes.

The cell lines analyzed above are derived from different origins and the differences may be due to cell type specificity. Therefore we employed FaDu cell lines, with or without over-expression of the HIF1a gene, to analyze differentially methylated genes in an isogenic background. The expression of HIF1a induces cells to become invasive. In this case, the overall methylation patterns of these two cell lines look very similar. However, closer examination of the differentially methylated genes that were identified showed that they are involved in the hypoxia response, tumorigenesis and metastasis; this was done using a literature search (see Table 1).

### 2.4. Correlation of the Methylome Domain Pattern with the Histone Modification Landscape

We also analyzed the relationship between histone modifications in the MCF-7 genome with the methylome domains using ChIP-chip. The results showed that H3K9Ac was organized in domains and that these domains correlated with the methylated zones in MCF-7 (Figure 4). H3K27Me3 modifications were also organized in domains in the hypomethylated domains and this pattern was complementary to that of H3K9Ac. H3K9Me2 and H3K4Ac, as well as the heterochromatic HP1 protein, appear not to play a major role in the MCF-7 genome as the majority of their binding signals and positions were similar to that of the control antibody in the control ChIP-chip analysis (data in supplementary).

### 2.5. An *en-bloc* Change in the Methylation Domains after Treatment with TSA

**Figure 5.**
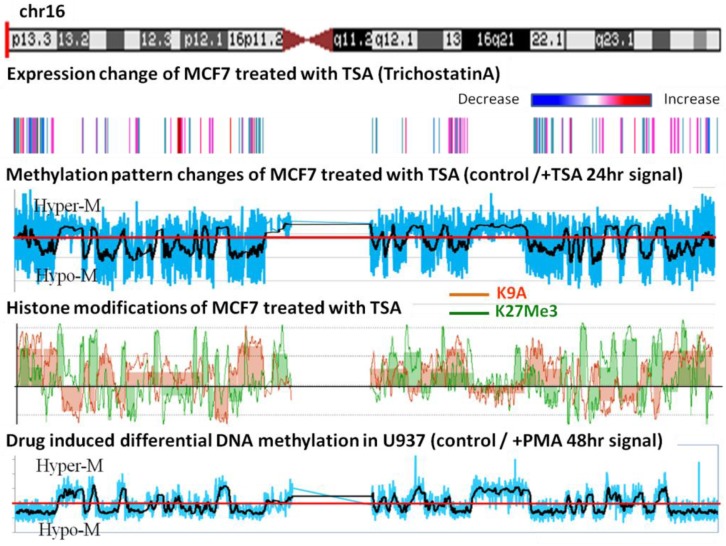
Changes in methylation pattern after drug treatment. Top panel, chromosome 16. Second panel, transcriptome data from the Affymetric analysis of MCF-7 with the level of transcription from high (red) to low (blue). Third panel, differential methylation changes in the MCF-7 genome after treatment with TSA; the blue line represents the ratio of methylation before and after drug treatment and the black line represents the statistical average for the data. Fourth panel, histone modification changes after TSA treatment. *En-bloc* changes similar to the methylation changes are observed. Fifth panel, changes in methylation pattern of U937 cells before and after the induction of cell differentiation by PMA.

In order to see whether the hypomethylated domains behave as units during environmental perturbations, we treated MCF-7 with trichostatin A, which is an HDAC inhibitor [14,15]. We divided the array-CGH data from the control by the data of TSA treated samples and plotted the ratio; the average of the ratio is shown in black smooth lines. The ratios above one indicate that DNA became hypermethylated after TSA treatment and *vice versa*. An example of chromosome 16 is shown in Figure 5. This treatment caused an *en-bloc* change in the methylation of the epigenomic domains. Furthermore, when U937 cells were induced to differentiate by the treatment with PMA, they also exhibited almost identical genome-wide domain-based methylation changes (Figure 5, lowest panel). These results show that the identified methylation domains seem to be the unit within the genome that responds to external perturbations.

### 2.6. Correlation between Histone Modification, DNA Methylation and Gene Expression

**Figure 6.**
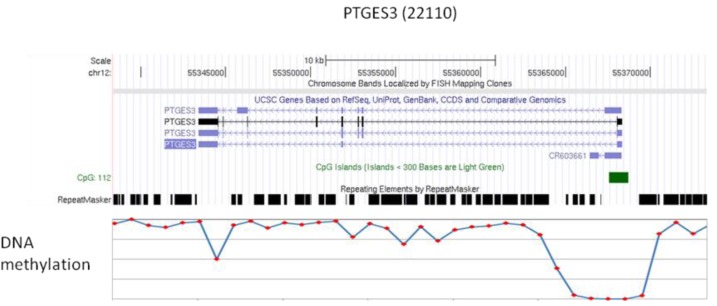
An example of a highly expressed gene with promoter hypomethylation and gene body hypermethylation. Top panel is the gene map of PTGES3 with the expression number in parenthesis. The lower panel is the level of methylation as determined by bisulfite sequencing; the transcription is from right to left.

**Figure 7.**
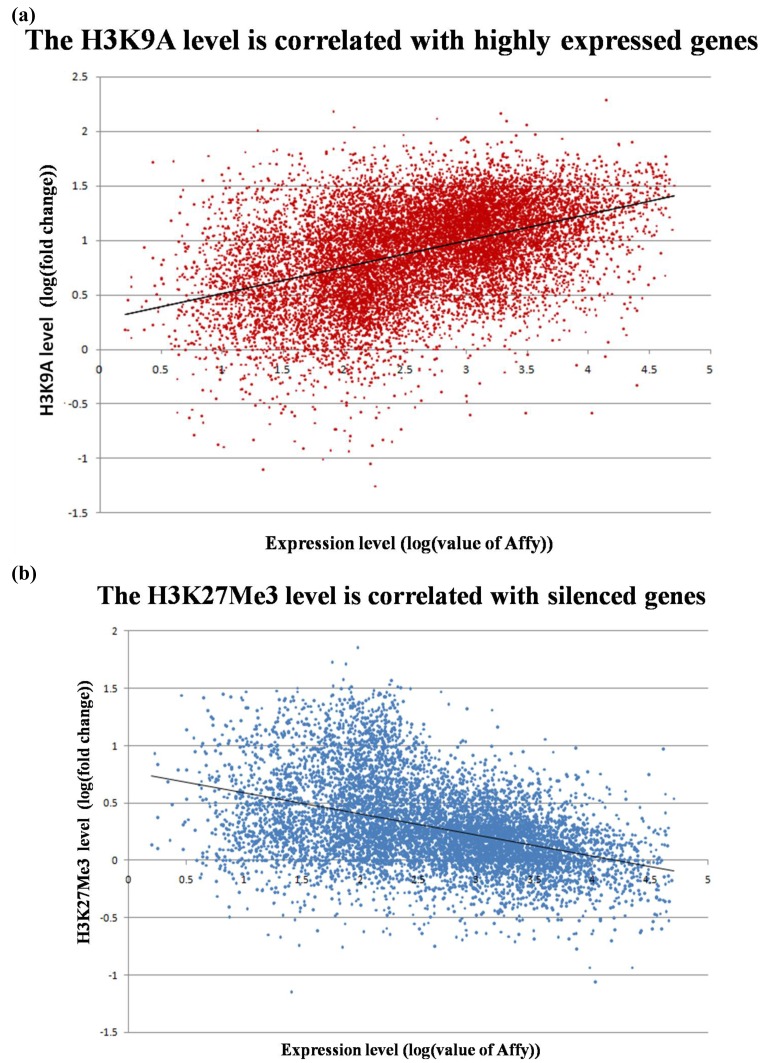
Correlation between histone modification and gene expression. Gene expression data are from a microarray analysis. (**a**) Positive correlation between H3K9Ac modification and gene expression (r = 0.546; *p* < 0.01); (**b**) Negative correlation between H3K27Me3 modification and gene expression (r = −0.368; *p* < 0.01). Statistical analysis and Pearson correlation coefficient was carried out using SPSS15.0.

**Figure 8.**
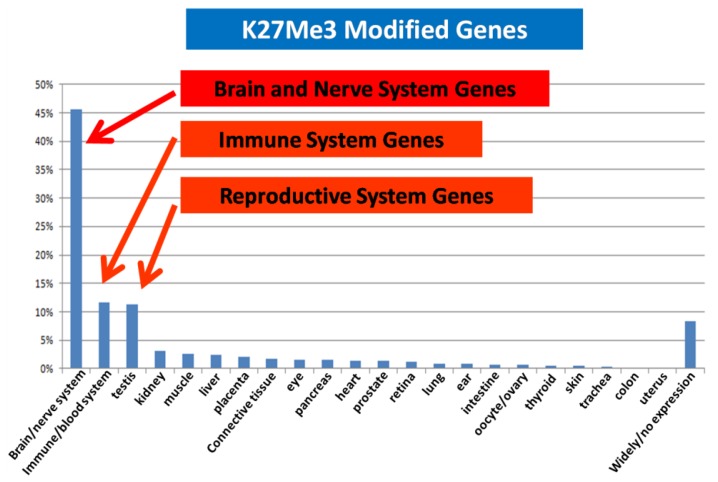
H3K27Me3 modification in the MCF-7 genome is enriched for brain, immune and reproductive genes.

We compared the epigenetic modifications identified in this study with the gene expression patterns obtained from an Affymetric analysis of MCF-7 cells and found that actively expressed genes were associated with promoter demethylation and intragenic methylation (see Figure 6 for example). H3K9Ac was also associated with active expression (Figure 7a), whereas H3K27Me3 was correlated with low gene expression (Figure 7b). Upon close examination we found that the majority of transcribed genes have H3K9Ac only in the 5′ region, whereas very highly expressed genes, such as ribosomal protein genes, have H3K9Ac in both their promoter and intragenic regions. In contrast, silenced genes were associated with promoter methylation, intragenic demethylation and the presence of H3K27Me3. An analysis of the silenced genes associated with H3K27Me3 showed that they are highly enriched for brain, immune and developmentally regulated genes (Figure 8).

## 3. Discussion

### 3.1. Epigenomic Domain Organization of Tumor Genomes

In this report we describe how we have developed a rapid high throughput McrBC-array method for scanning the methylome pattern of tumor genomes. We validated the technique by direct bisulfite sequencing of randomly chosen sites and the whole MCF-7 genome. Although the coverage of CpG sites in MCF-7 genome bisulfite sequencing is only 85% at this time, the methylation domain pattern obtained is the same as that obtained by the McrBC and TspRI-ExoIII techniques. We believe the McrBC-array method is inexpensive and rapid, with a high throughput that allows the discovery of genes or regions that are altered in the tumor genomes in terms of their DNA methylation. Since tumors are heterogeneous, and probably vary significantly between individuals, a high throughput comparative analysis of many tumors is likely to be necessary to deduce the common epigenetic alterations associated with a specific tumor type or with a specific stage of cancer. Although high throughput sequencing is able to analyze genome-wide methylation at a nucleotide resolution, the method is very expensive and difficult to carry out when there are large numbers of tumor samples. We believe the McrBC-array method described in the present work is a useful and inexpensive way of carrying out comparative methylome analysis of a large number of tumor genomes. Using this approach, it will be possible to analyze the differential methylation of cancers in a systematic way as we have done between the genomes of invasive and non-invasive tumor cell lines.

Cytosine methylation represents one of the surface codes found on DNA, which include the unused hydrogen bonds of base-pairs, the methyl group from thymidine, the backbone phosphate group, hydration water molecules and the codes from various histone modifications. These vast complicated surface codes are required for the highly plastic interactions that occur with protein and RNA regulators in the cell in order to achieve context-dependent regulation of gene expression. Because of its association with primary DNA sequence, CG methylation is used as context-acquired memory with an intermediate half-life that allows subsequent fixation of the genotype during evolution. In contrast, the on-and-go histone modifications represent a highly plastic short half-life response that allows the immediate contingent modification of gene expression. Thus it is interesting that DNA methylation and histone modifications are both organized in similar domains; this suggests a master mechanism for such organization. We are now investigating the dynamic process involved in these epigenetic changes as well as their kinetics using perturbations by drugs.

### 3.2. A Thrifty Hypothesis for Genome-Wide Hypomethylation in Tumor Genomes

Our analysis shows that there are common hypomethylated domains in a wide range of tumor genomes. It is interesting that these hypomethylated domains are mainly located in gene deserts or gene poor regions of the human genome that are associated with large genes that express mainly in the neural, immune and reproductive systems. These regions apparently are not needed by tumor cells for growth and survival. We speculate that the reason for the demethylating of these low CpG domains, in these gene deserts or in regions with few tissue-specific or developmental genes, is because such demethylation of the DNA in these domains results in the tumor cells saving energy and resources. Part of these saved resources would be those needed for maintaining the methylation of DNA in these domains. Overall, the changes in methylation would seem to support thrifty tumor genome hypothesis. The subtle variations in methylation in the hypomethylated domain between tumor genomes detected in this study may represent recruitment of genes specifically needed by each different tumor type in a particular cell or tissue context. Thus a detailed study of the genes that show variation in methylation between tumors may provide clues that will help our understanding of the context-dependence of the tumorigenic process. In fact we have already identified a number of tumor suppressor genes that show subtle variations in methylation across the various hypomethylated domains. Analysis of the isogenic FaDu cell lines with or without HIF1a, which results in differences in invasion capability, indeed shows differential methylation of many genes that are associated with metastasis (Table 1). Another consequence of demethylation in the epigenomic domains is that repetitive sequences in these domains may become activated for transcription, transposition or recombination. The deregulation of these interspersed repeat elements may result in genome instability as well as alterations in global gene expression program. This is likely to have carcinogenic consequences [16].

### 3.3. *En-bloc* Methylation Changes within the Methylation Domain: Implication in Terms of a Genome Domain cis-Regulator and trans-Regulatory Mechanism

Our results suggest that human genome is organized into epigenomic domains containing different group of genes. Such an organization may facilitate the higher order regulation of genome-wide gene expression through *en-bloc* control of epigenetic modifications. Our analysis of cells treated with drugs indeed supports such a model. The domain organization implies that there are cis- and trans-regulators that govern the activity of these domains. Deducing the regulatory mechanism of the epigenomic domains would allow us to understand how higher order regulation of genome-wide expression occurs. Our preliminary analysis of the temporal changes in DNA methylation during drug treatment indeed showed that all-or-none allelic *en-bloc* changes in CG methylation occur. This suggests that there are methylation boundary elements that govern the methylation status of the entire domain. Examination of the hypomethylated domains also shows that they are enriched for newly evolved human sequences when compared with the chimpanzee genome (manuscript in preparation). This result may indicate that these hypomethylated domains may represent units that are involved in genome evolution perhaps independent of other areas within the genome.

### 3.4. Correlation of Gene Expression with Epigenetic Modifications

Our analysis shows that, generally, many genes in the hypomethylated domains show relatively low levels of expression. This seems to be contrary to the current concept of gene activation when a promoter is demethylated. However, a closer examination showed that the intron-containing genes in these domains are hypomethylated in the gene body but hypermethylated in the promoter region, which is in contrast to the active genes where there is hypomethylation of the promoter and hypermethylation of the gene body (see Figure 6 and [17-20]). Since the gene body is much larger than the promoter region, the overall methylation of the silenced genes will, on average, be much lower than that of the active genes. This accounts for these silenced genes being present in the hypomethylated domains. However, the patterns of methylation in some genes are rather complicated with a number of large genes showing mosaic type methylation where alternating methylation regions are interspersed with methylated regions. The biological meaning of such complex intragenic methylation patterns is unknown at the present time and requires further investigation. We also observed that some are genes fully methylated but are still expressed, and that some genes with full methylation can be rescued by histone acetylation.

It is interesting that these highly transcribed genes are associated with H3K9Ac in both the promoter and the gene body, whereas the lower expressed genes are associated with H3K9Ac only in the 5′ region. This observation suggests that H3K9Ac in the promoter facilitates transcription initiation, whereas intragenic modification facilitates transcription elongation. Similarly, intragenic association with H3K27Me3 should inhibit transcription elongation and thus insure permanent silencing of the gene, whereas association of H3K27Me3 in the promoter region may represent only a temporary silencing of the gene. We found that the genes silenced in association with H3K27Me3 are enriched for brain, immune and reproductive genes. These genes apparently are not needed by MCF-7 breast cancer cells and therefore are silenced to save the cell's resources.

### 3.5. Implication in Cancer Therapy

The presence of an epigenomic domain organization within a range of tumor genomes suggests the presence of master regulators that control the epigenetic modifications of these domains and thus gene expression. Targeting of these master regulators may offer a novel target for cancer therapy involving the reversing of this epigenomic organization back towards the situation found in normal cells. We are currently engaged in a search for such master regulators in tumor cells.

## 4. Experimental Section

### 4.1. Cell Culture

The MCF-7, CL1-0 and CL1-5 cells were cultured in RPMI1640 medium (GIBCO/BRL) supplemented with 10% (v/v) fetal bovine serum (GIBCO/BRL), 2.0 g/L sodium bicarbonate. The MDA-MB-231 cells were cultured in L15 medium (GIBCO/BRL) supplemented with 10% (v/v) fetal bovine serum (GIBCO/BRL). The FaDu, FaDu-HIF1α, HepG2, Huh7 and Mahlavu cell lines were cultured in Dulbecco's Modified Eagle's Medium (GIBCO/BRL). All cell lines were incubated in a humidified 37 °C incubator with 5% CO_2_.

### 4.2. Genomic DNA Extraction

Cells were washed with 1× PBS and resuspended with cell lysis buffer. Cells were treated with 0.1 mg/mL of RNaseA for an hour at 37 °C and 0.3 mg/mL proteinase K for 12–16 hours at 55 °C. Samples were extracted with an equal volume of phenol/chloroform/isoamyl alcohol mixture (24:25:1). The extraction procedure was repeated until the interface is clean. An equal volume of chloroform was then added and the mixture centrifuged for 10 minutes at 13000× g. Finally, the aqueous phase was removed and precipitated with ethanol. After removal of the supernatant, the DNA pellet was washed with 70% ethanol, air-dried, and dissolved in triple distilled H_2_O. The integrity of the DNA extracted was checked by 1.2 % (w/v) agarose gel electrophoresis. The concentration of DNA was estimated by ultraviolet spectrophotometry.

### 4.3. Human Tissue Genomic DNA

Human genomic DNA from normal brain, breast, liver, testis, leukocytes, as well as breast tumors were purchased from BioChain.

### 4.4. Restriction Enzyme Digestion of Genome DNA

After quantification by UV absorbance, 1 μg of genome DNA sample was digested overnight with 2.5–10 units of restriction enzyme(s) in a 30 μL reaction. The conditions of each restriction enzyme digestion, including the temperature and the buffers, were as recommended in the manufacturer's protocol.

### 4.5. Preparation of RNA

Cells were rinsed twice with 1× PBS. Total RNA was extracted according to the RNeasy® Mini Kit Spin Protocol (QIAGEN). The integrity of the RNA extract was checked by agarose gel electrophoresis and the concentration of RNA was estimated by ultraviolet spectrophotometry.

### 4.6. Extraction of Large Fragments after Digestion with McrBC

A total of 25–30 μg of DNA was digested with McrBC, which cleaves DNA containing methylcytosine; this was done in a 100 μl total volume solution for 3hrs. The DNA was then separated using a low percentage agarose gel. The uncut large fragments were recovered from the appropriate gel fragment using a QIAquick® Gel Extraction Kit and the Instruction Manual (QIAGEN).

### 4.7. Chromatin Immunoprecipitation (ChIP) Assay

The ChIP assay was performed according to the manufacturer's protocol (Upstate Biotechnology, Inc., Lake Placid, NY, USA) except that the sonication conditions were changed to 48 rounds, each of 5 seconds of sonication at 50% power output, with a 40 seconds rest between rounds. Specific antibodies were used for the immunoprecipitation. After the protein-DNA cross-links in the immunoprecipitation complex were reversed, the DNA was extracted for subsequent PCR analysis or array CGH analysis. The PCR products were examined by agarose gel electrophoresis.

### 4.8. Array-CGH Protocols

Array-CGH analysis was performed using the Agilent Human Genome CGH Microarray 185 K, 244 K and 1M (Agilent. Technologies) high resolution 60-mer oligonucleotide-based microarray sets containing 184672, 243431 and 963029 probes, respectively. Labeling and hybridization were performed according to the protocol provided by Agilent. MCF-7, MDA-MB-231 and the genomes from the various tissues were processed as shown in Figure 1; HpaII was selected as the methylation-sensitive restriction enzyme. Briefly, 2–3 μg of the undigested and digested DNA were double-digested with AluI and RsaI for 2 hours at 37 °C. The digested DNA was labeled by random priming using the Agilent Genome DNA Labeling Kit Plus. ExoIII-digested and control mock-digested DNA were pooled and hybridized with Human Cot I DNA at 65 °C. Washing was performed according to the Agilent protocol. The arrays were analyzed using the Agilent DNA Microarray Scanner AA and Agilent Feature Extraction software (v.9.1). The results are presented using Agilent CGH Analytics software (v.3.4). The Cy3 hybridization intensity was normalized to Cy5 for comparison among the samples. The log2 ratios (log2 Cy5/Cy3) were calculated and compared. The array-CGH data, including raw data and normalized data, have been deposited at the NCBI GEO website (GEO accession no. GSE9015).

### 4.9. Analysis of Array-CGH Data

The analysis of the array-CGH data was done using the program Genomic Workbench Standard Edition 5.0.14.

### 4.10. Affymetrix Analysis

Affymetrix microarray was performed using Human U133 2.0 (Affymetrix). The details of the methods related to RNA quality, sample labeling, hybridization and expression analysis conformed to the manual of the Affymetrix Microarray Kit. The microarray data has been deposited at the NCBI GEO website (GEO accession number GSE9015).

### 4.11. Bioinformatics Analysis of the Bisulfite Sequencing Data

There are 392,998,582 reads from ten lanes of Solexa sequencing data. The read length of the Solexa sequencing is 100 bp. We used the mapping program BS seeker [1] to align the bisulfite sequencing reads against the human genome (UCSC HG18) allowing a maximum three mismatches. A positive mapping of a bisulfite read is considered as a read that forms a uniquely hit on the human genome. On average, uniquely aligned reads with mismatches of less than three base pair form 76.73% of all reads, covered 70.72% of the human genome and involve 86.64% of all CpG sites. We next used an in-house Java program to calculate the methylation and non-methylation CpG density from the positive mapping results.

### 4.12. Recovery of DNA from Agarose Gel

After analyzing the PCR products by agarose gel electrophoresis, the piece of gel containing the desired DNA fragment was physically removed with a scalpel. The DNA was then recovered from the gel fragment according to a QIAquick® Gel Extraction Kit and by following the Instruction Manual (QIAGEN).

## 5. Conclusions

In conclusion, we believe that dysregulation of the master control of the epigenomic domains results in genome-wide changes in the epigenetic modifications within a gene and that is able to maximize cell survival and proliferation. These changes have consequences in terms of alterations in cell differentiation gene expression program, with respect to gene mutations and in terms of DNA recombination, with the latter resulting in chromosome aberrations. These epigenomic domain master regulators may provide effective therapeutic targets for the treatment of cancer.

## Figures and Tables

**Table 1.** Genes differentially methylated between the FaDu and FaDu-HIF1a cell lines.

**Category**	**Gene name**
Metastasis related genes	LMNA, VANGL, RGS4, MALL, DLX2, TNS1, WNT7A, CXCL9, EREG, MAD2L, KIF2A, SPRY4, FOXO3, SEMA3C, ADAM9, SNAI2, FABP5, EDG2, CXCL12, IFIT2, SLK, FOSL1, MMP7, PVRL1, JAM2, JAM3, VDR, JUB, LGALS3, HIF1a, LASP1, CCR7, HOXB2, ITGA3, SOX9, SMAD4, SNF1LK, MID1, KLF8, MSN
Tumor Suppressor genes	RPRM, PTPRG, SULF1, MTAP, RASEF, SYK, PDCD4, IFT88, INTS6, COPS2, ADAMTS18, CDH13, STARD8
Tumorigenesis genes (oncogene, overexpression, prognosis markers)	JUNB, ERG(TF), PBX1(TF), REL, ATF2(TF), H2AFZ, CARD6, SPZ1 (TF), DRD1, ICK, SDK1(FRA7B), DMD, ETV1, JAZF1, DBF4, TRPV6, DEFB1, STC1, HNF4G, RAD21, MYC, DOCK8, RLN1,2, MPDZ, KLF9, PTGS1, TSG101, CRY1, GPC5, HBLD1, UBE2Q2, SS18, KLK2,3, MAGEH1, MAGEA4, LUZP4
Apoptosis related genes	BHLHB, PDCD4, PDCD5, BAX. GZMH, FEM1B
Hypoxia response genes	JMJD1A, EIF2AK3, IGFBP5, FOXP1, CXCL9, CTGF, NKX3-1, TXN, HIF1a, NOS2A, KLF2
Cell cycle genes	MAD2L, CCNG1, DBF4, SMC2, VRK1, RASGR, PCNA

## Supplementary Files

Supplementary File 1 PDF-Document (PDF, 6427 KB)
