# Supplementary material for: Common Altered Epigenomic Domains in Cancer Cells: Characterization and Subtle Variations
Source: Cancers (Basel). 2011 Apr 18;3(2):1996–2013. doi: 10.3390/cancers3021996 (PMC3757401; doi:10.3390/cancers3021996)
Supplement: Supplementary File 1 — PDF-Document (PDF, 6427 KB) [file cancers-03-01996-s001.pdf]

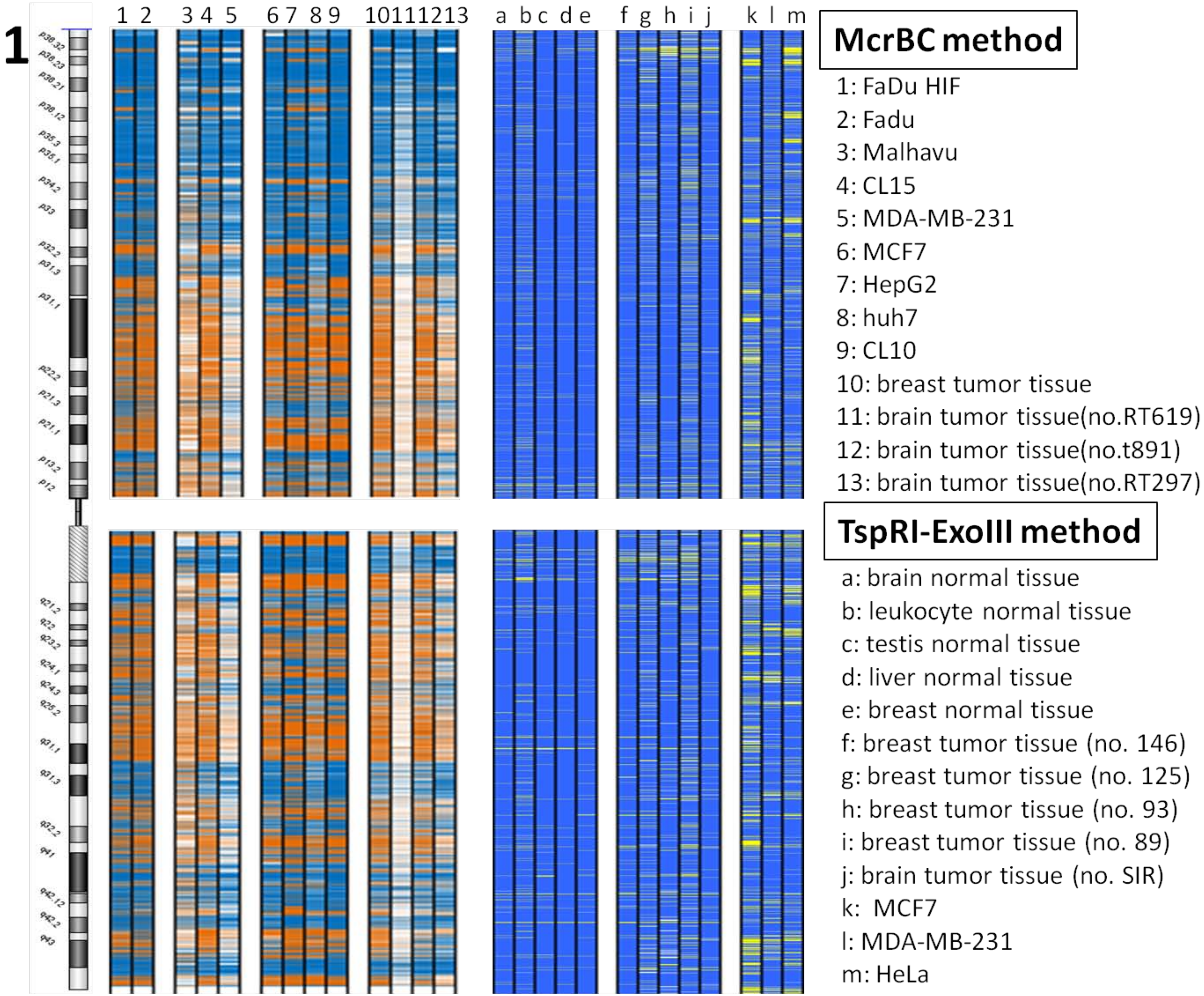
1
1 2 3 4 5 6 7 8 9 10 11 12 13
a b c d e f g h i j k l m
McrBC method
1: FaDu HIF
2: Fadu
3: Malhavu
4: CL15
5: MDA-MB-231
6: MCF7
7: HepG2
8: huh7
9: CL10
10: breast tumor tissue
11: brain tumor tissue(no.RT619)
12: brain tumor tissue(no.t891)
13: brain tumor tissue(no.RT297)
TspRI-ExoIII method
a: brain normal tissue
b: leukocyte normal tissue
c: testis normal tissue
d: liver normal tissue
e: breast normal tissue
f: breast tumor tissue (no. 146)
g: breast tumor tissue (no. 125)
h: breast tumor tissue (no. 93)
i: breast tumor tissue (no. 89)
j: brain tumor tissue (no. SIR)
k: MCF7
l: MDA-MB-231
m: HeLa

2

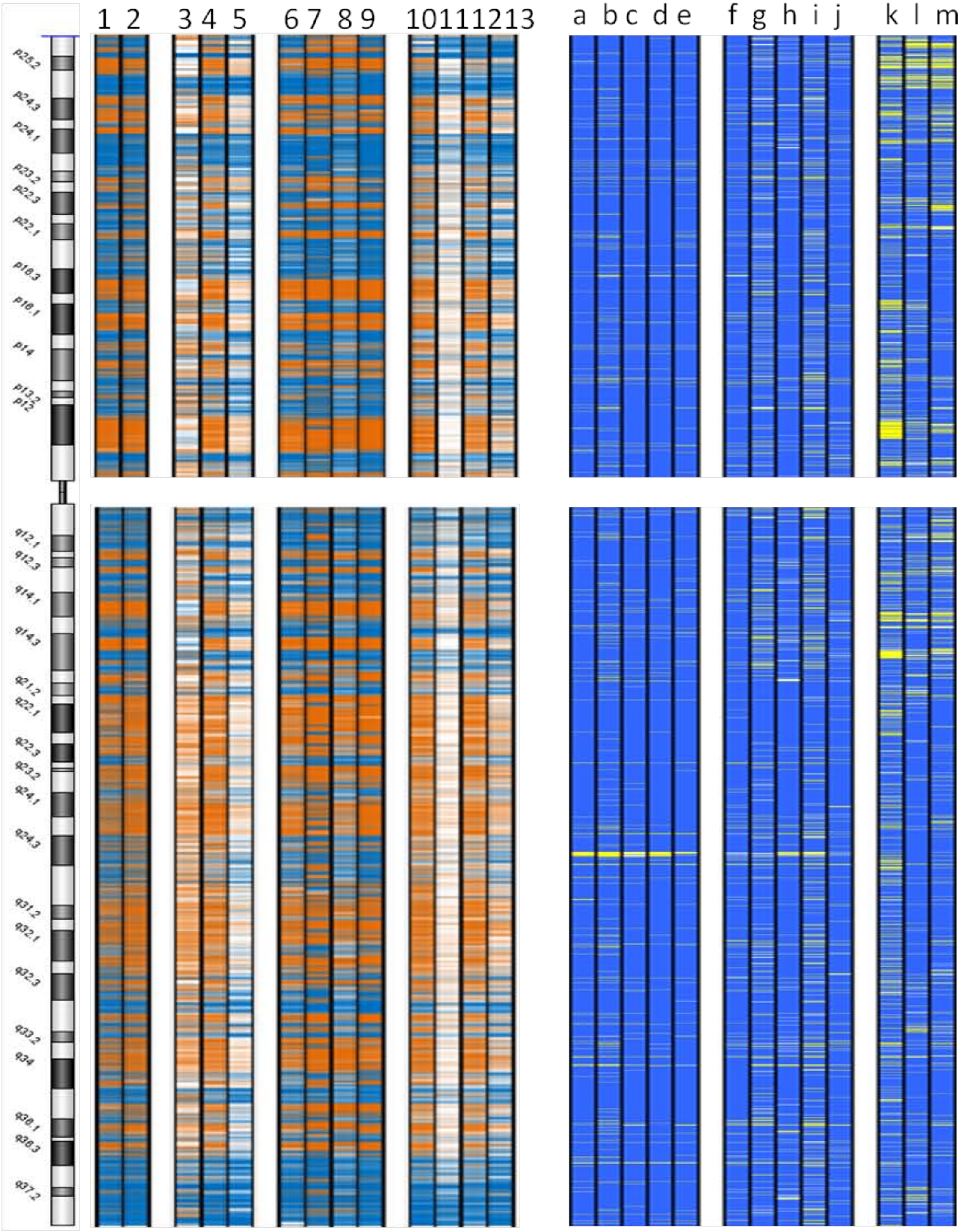

McrBC method

1: FaDu HIF
2: Fadu
3: Malhavu
4: CL15
5: MDA-MB-231
6: MCF7
7: HepG2
8: huh7
9: CL10
10: breast tumor tissue
11: brain tumor tissue(no.RT619)
12: brain tumor tissue(no.t891)
13: brain tumor tissue(no.RT297)

TspRI-ExoIII method

a: brain normal tissue
b: leukocyte normal tissue
c: testis normal tissue
d: liver normal tissue
e: breast normal tissue
f: breast tumor tissue (no. 146)
g: breast tumor tissue (no. 125)
h: breast tumor tissue (no. 93)
i: breast tumor tissue (no. 89)
j: brain tumor tissue (no. SIR)
k: MCF7
l: MDA-MB-231
m: HeLa

3

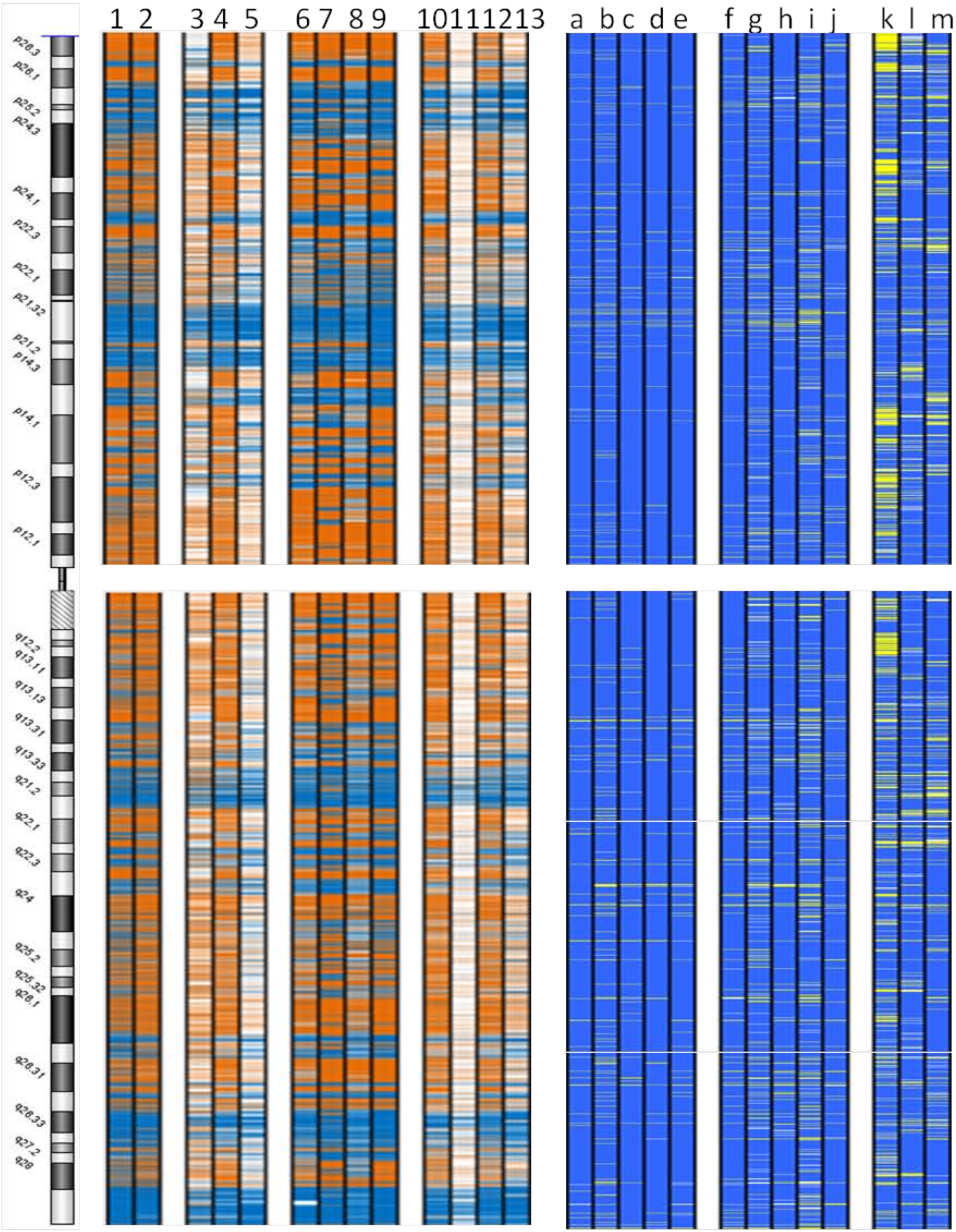

McrBC method

1: FaDu HIF
2: Fadu
3: Malhavu
4: CL15
5: MDA-MB-231
6: MCF7
7: HepG2
8: huh7
9: CL10
10: breast tumor tissue
11: brain tumor tissue(no.RT619)
12: brain tumor tissue(no.t891)
13: brain tumor tissue(no.RT297)

TspRI-ExoIII method

a: brain normal tissue
b: leukocyte normal tissue
c: testis normal tissue
d: liver normal tissue
e: breast normal tissue
f: breast tumor tissue (no. 146)
g: breast tumor tissue (no. 125)
h: breast tumor tissue (no. 93)
i: breast tumor tissue (no. 89)
j: brain tumor tissue (no. SIR)
k: MCF7
l: MDA-MB-231
m: HeLa

4

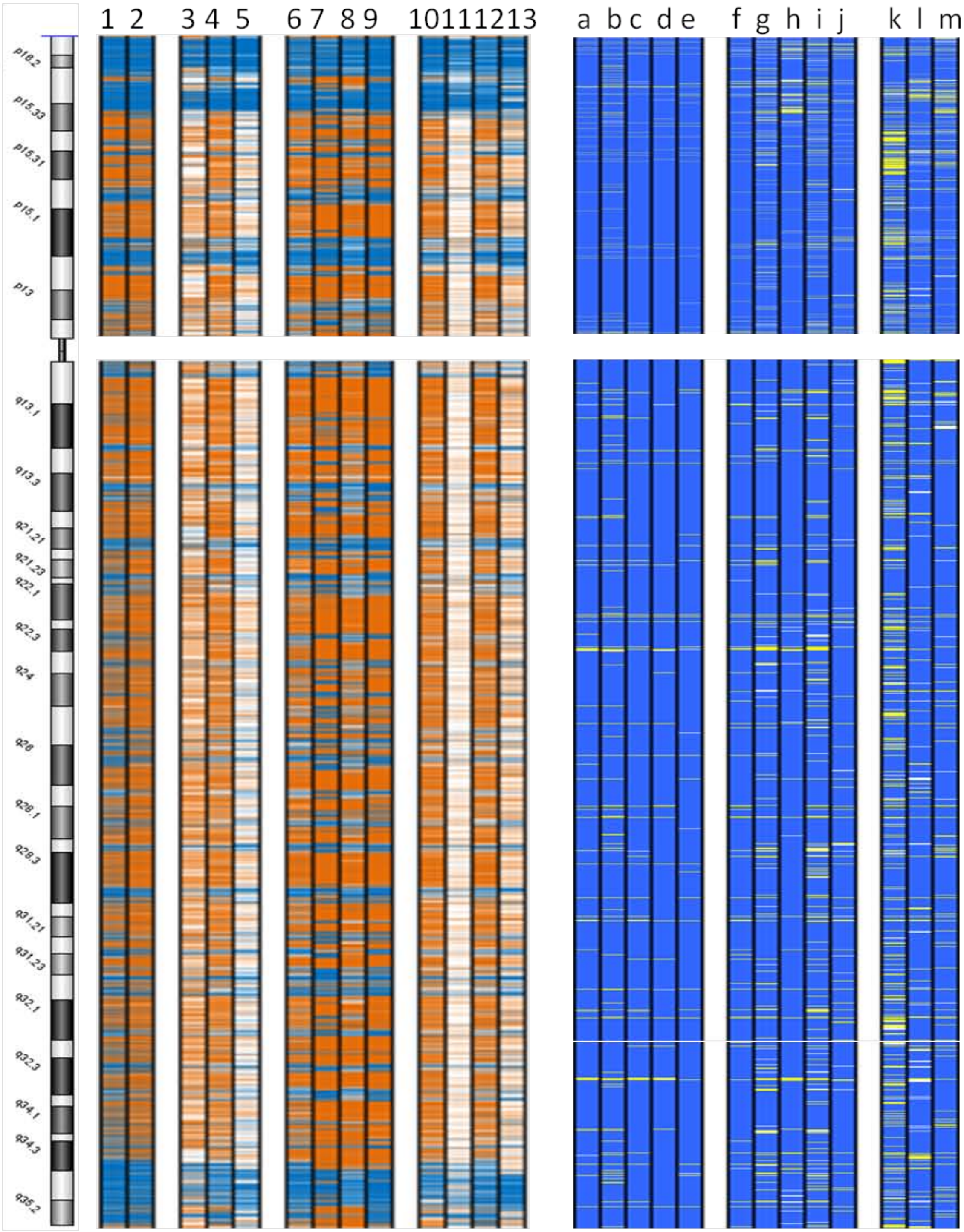

McrBC method

1: FaDu HIF
2: Fadu
3: Malhavu
4: CL15
5: MDA-MB-231
6: MCF7
7: HepG2
8: huh7
9: CL10
10: breast tumor tissue
11: brain tumor tissue(no.RT619)
12: brain tumor tissue(no.t891)
13: brain tumor tissue(no.RT297)

TspRI-ExoIII method

a: brain normal tissue
b: leukocyte normal tissue
c: testis normal tissue
d: liver normal tissue
e: breast normal tissue
f: breast tumor tissue (no. 146)
g: breast tumor tissue (no. 125)
h: breast tumor tissue (no. 93)
i: breast tumor tissue (no. 89)
j: brain tumor tissue (no. SIR)
k: MCF7
l: MDA-MB-231
m: HeLa

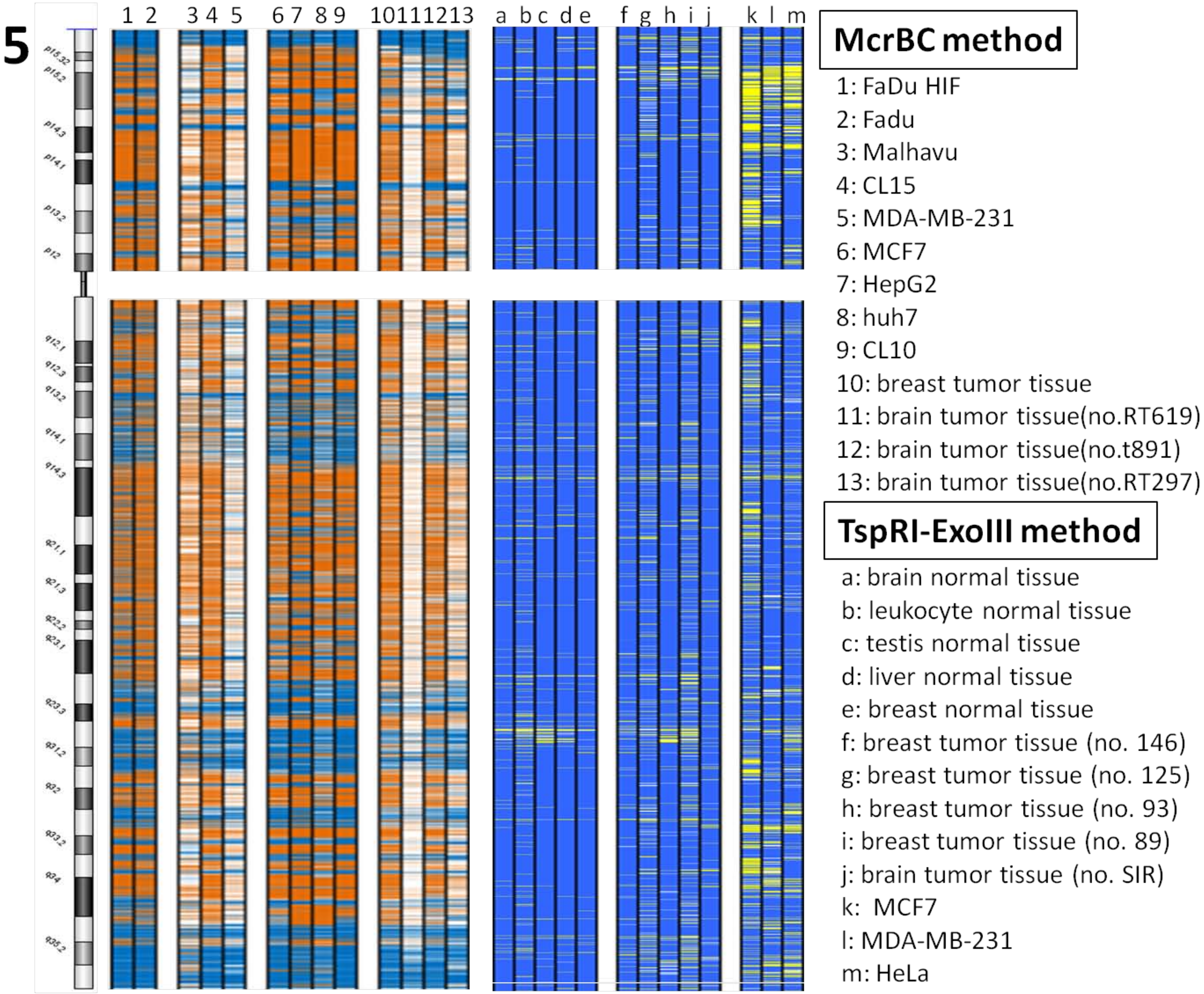
5
1 2 3 4 5 6 7 8 9 10111213 a b c d e f g h i j k l m
McrBC method
1: FaDu HIF
2: Fadu
3: Malhavu
4: CL15
5: MDA-MB-231
6: MCF7
7: HepG2
8: huh7
9: CL10
10: breast tumor tissue
11: brain tumor tissue(no.RT619)
12: brain tumor tissue(no.t891)
13: brain tumor tissue(no.RT297)
TspRI-ExoIII method
a: brain normal tissue
b: leukocyte normal tissue
c: testis normal tissue
d: liver normal tissue
e: breast normal tissue
f: breast tumor tissue (no. 146)
g: breast tumor tissue (no. 125)
h: breast tumor tissue (no. 93)
i: breast tumor tissue (no. 89)
j: brain tumor tissue (no. SIR)
k: MCF7
l: MDA-MB-231
m: HeLa

6

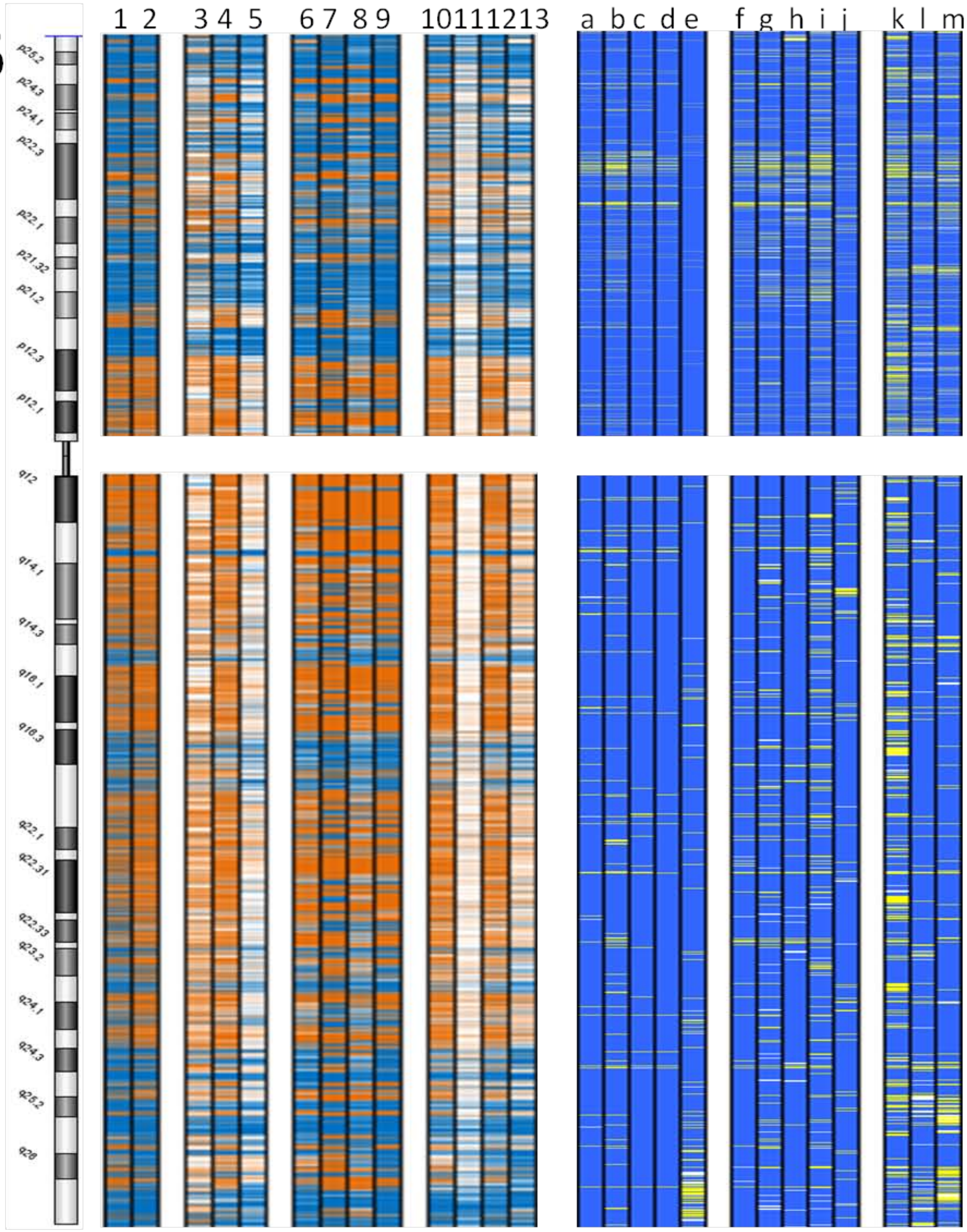

McrBC method

1: FaDu HIF
2: Fadu
3: Malhavu
4: CL15
5: MDA-MB-231
6: MCF7
7: HepG2
8: huh7
9: CL10
10: breast tumor tissue
11: brain tumor tissue(no.RT619)
12: brain tumor tissue(no.t891)
13: brain tumor tissue(no.RT297)

TspRI-ExoIII method

a: brain normal tissue
b: leukocyte normal tissue
c: testis normal tissue
d: liver normal tissue
e: breast normal tissue
f: breast tumor tissue (no. 146)
g: breast tumor tissue (no. 125)
h: breast tumor tissue (no. 93)
i: breast tumor tissue (no. 89)
j: brain tumor tissue (no. SIR)
k: MCF7
l: MDA-MB-231
m: HeLa

7

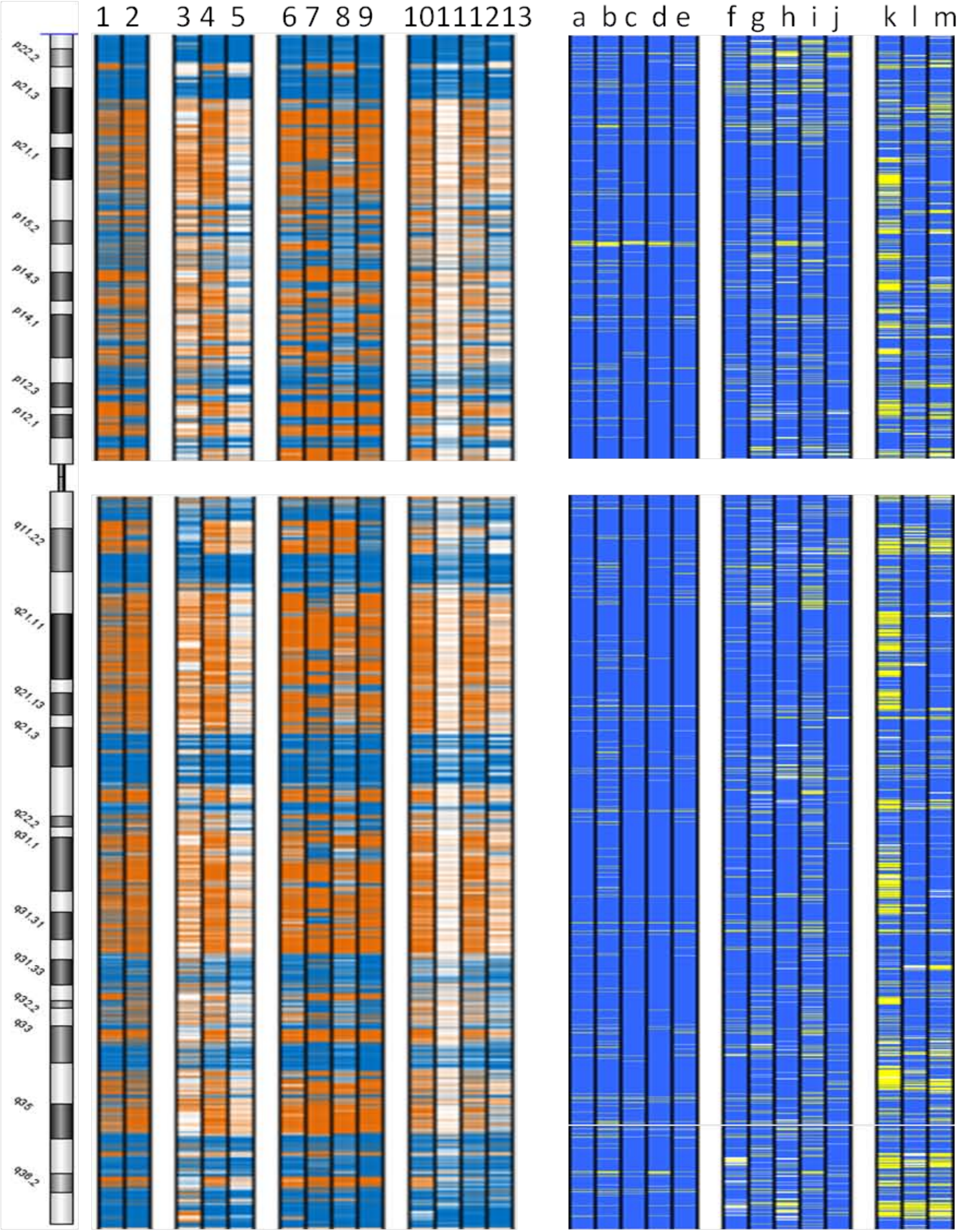

McrBC method

1: FaDu HIF
2: Fadu
3: Malhavu
4: CL15
5: MDA-MB-231
6: MCF7
7: HepG2
8: huh7
9: CL10
10: breast tumor tissue
11: brain tumor tissue(no.RT619)
12: brain tumor tissue(no.t891)
13: brain tumor tissue(no.RT297)

TspRI-ExoIII method

a: brain normal tissue
b: leukocyte normal tissue
c: testis normal tissue
d: liver normal tissue
e: breast normal tissue
f: breast tumor tissue (no. 146)
g: breast tumor tissue (no. 125)
h: breast tumor tissue (no. 93)
i: breast tumor tissue (no. 89)
j: brain tumor tissue (no. SIR)
k: MCF7
l: MDA-MB-231
m: HeLa

8

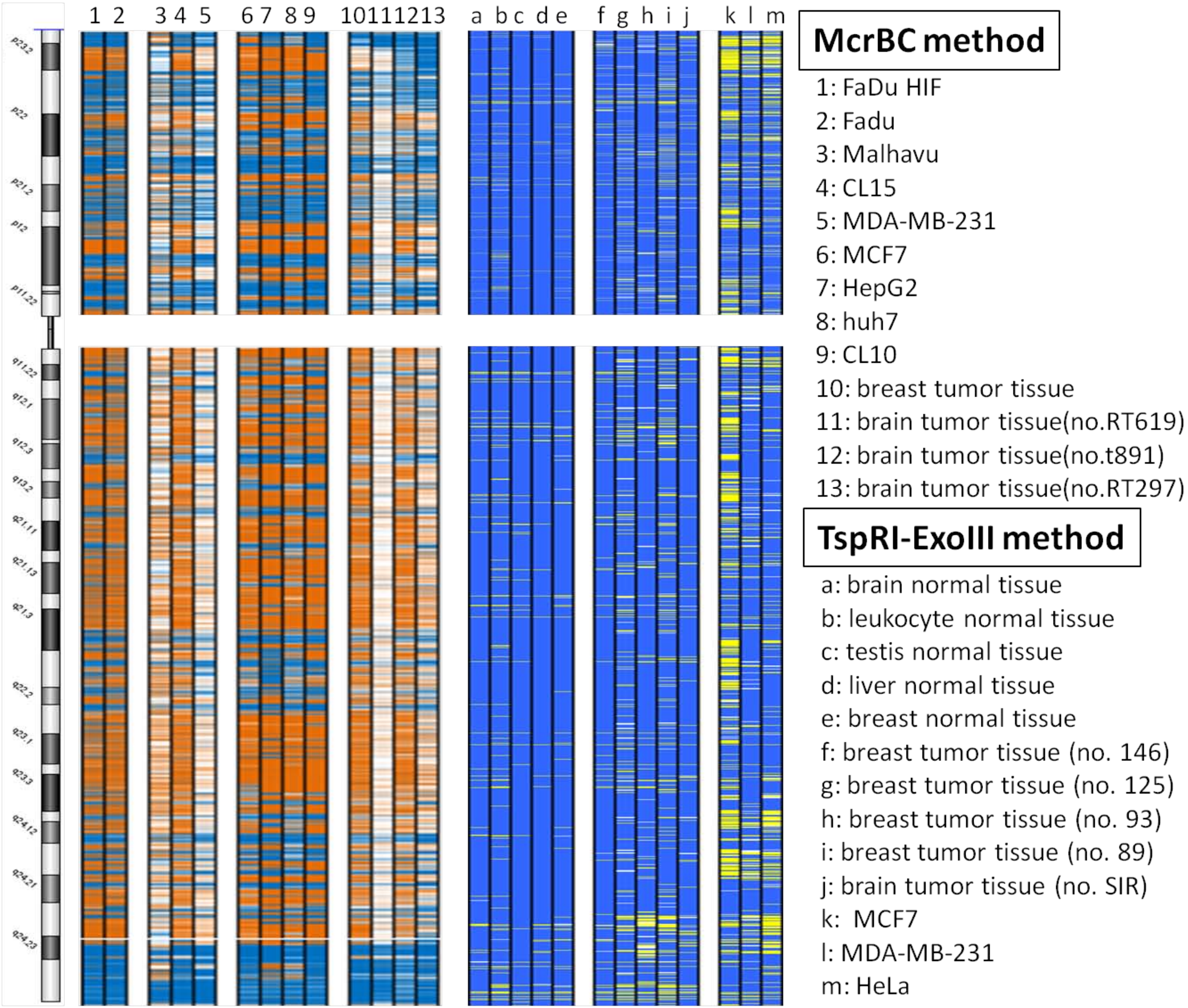
1 2 3 4 5 6 7 8 9 10 11 12 13
a b c d e f g h i j k l m
McrBC method
1: FaDu HIF
2: Fadu
3: Malhavu
4: CL15
5: MDA-MB-231
6: MCF7
7: HepG2
8: huh7
9: CL10
10: breast tumor tissue
11: brain tumor tissue(no.RT619)
12: brain tumor tissue(no.t891)
13: brain tumor tissue(no.RT297)
TspRI-ExoIII method
a: brain normal tissue
b: leukocyte normal tissue
c: testis normal tissue
d: liver normal tissue
e: breast normal tissue
f: breast tumor tissue (no. 146)
g: breast tumor tissue (no. 125)
h: breast tumor tissue (no. 93)
i: breast tumor tissue (no. 89)
j: brain tumor tissue (no. SIR)
k: MCF7
l: MDA-MB-231
m: HeLa

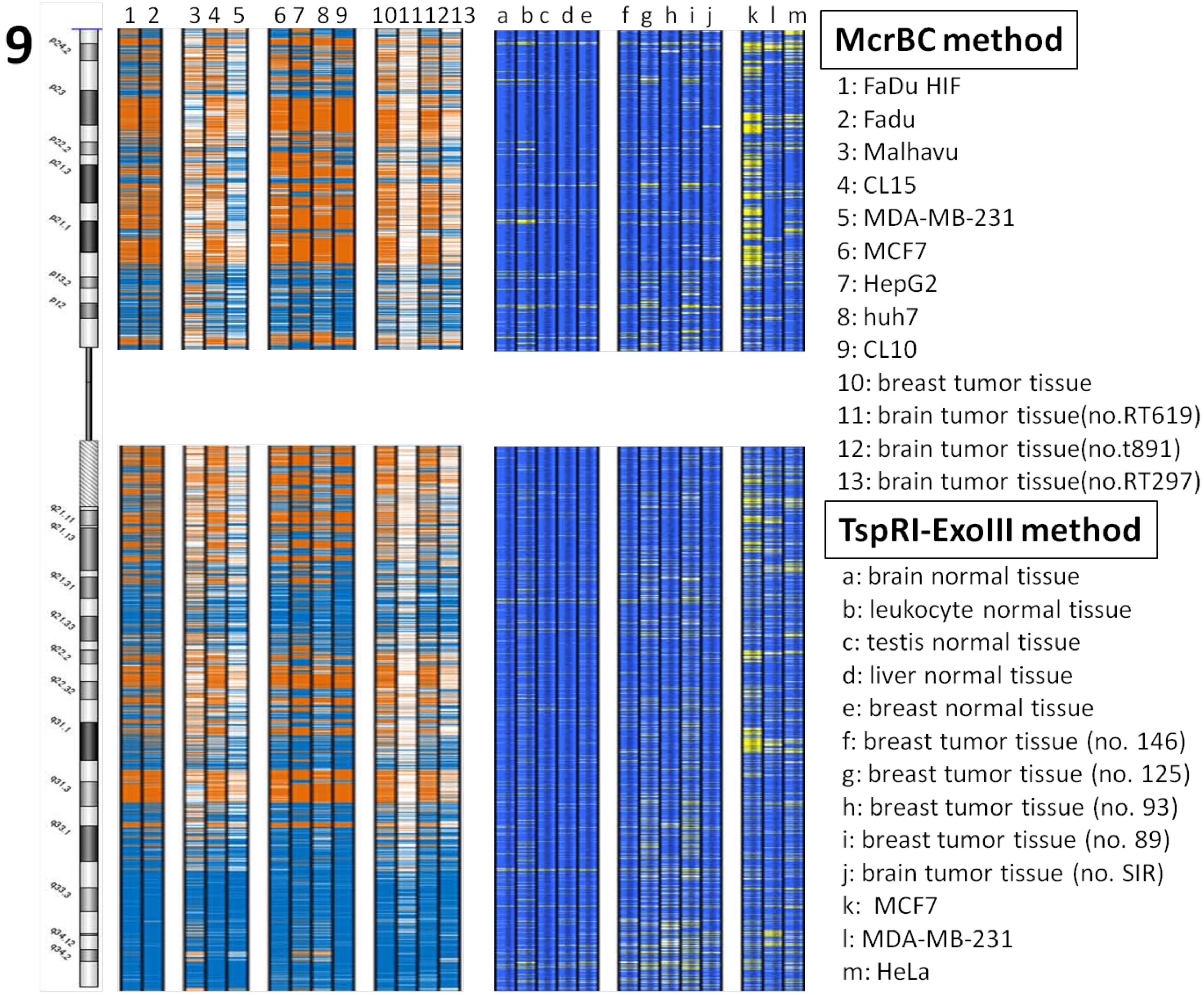
9
1 2 3 4 5 6 7 8 9 10 11 12 13
a b c d e f g h i j k l m
McrBC method
1: FaDu HIF
2: Fadu
3: Malhavu
4: CL15
5: MDA-MB-231
6: MCF7
7: HepG2
8: huh7
9: CL10
10: breast tumor tissue
11: brain tumor tissue(no.RT619)
12: brain tumor tissue(no.t891)
13: brain tumor tissue(no.RT297)
TspRI-ExoIII method
a: brain normal tissue
b: leukocyte normal tissue
c: testis normal tissue
d: liver normal tissue
e: breast normal tissue
f: breast tumor tissue (no. 146)
g: breast tumor tissue (no. 125)
h: breast tumor tissue (no. 93)
i: breast tumor tissue (no. 89)
j: brain tumor tissue (no. SIR)
k: MCF7
l: MDA-MB-231
m: HeLa

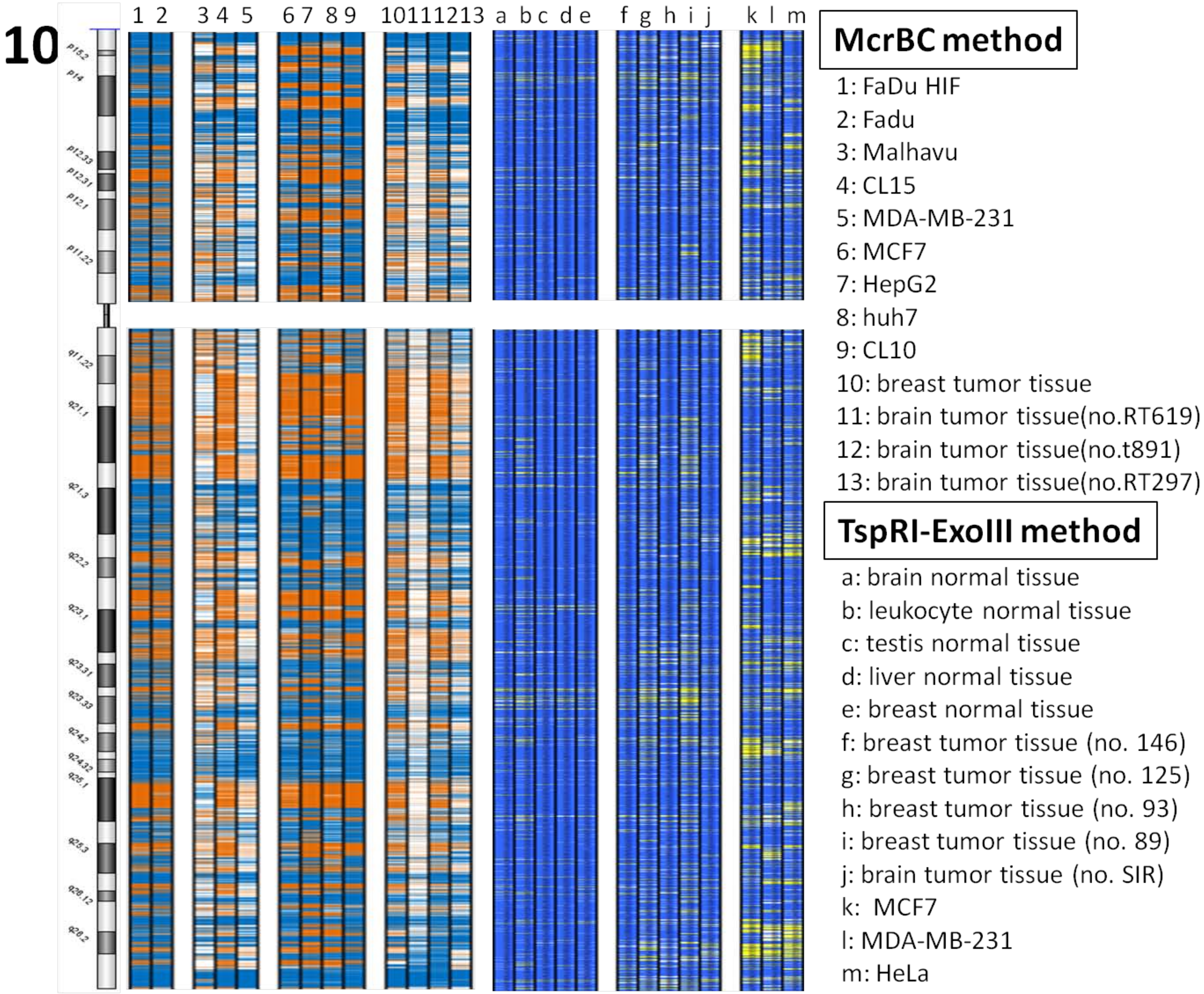
10
1 2 3 4 5 6 7 8 9 10 11 12 13 a b c d e f g h i j k l m
McrBC method
1: FaDu HIF
2: Fadu
3: Malhavu
4: CL15
5: MDA-MB-231
6: MCF7
7: HepG2
8: huh7
9: CL10
10: breast tumor tissue
11: brain tumor tissue(no.RT619)
12: brain tumor tissue(no.t891)
13: brain tumor tissue(no.RT297)
TspRI-ExoIII method
a: brain normal tissue
b: leukocyte normal tissue
c: testis normal tissue
d: liver normal tissue
e: breast normal tissue
f: breast tumor tissue (no. 146)
g: breast tumor tissue (no. 125)
h: breast tumor tissue (no. 93)
i: breast tumor tissue (no. 89)
j: brain tumor tissue (no. SIR)
k: MCF7
l: MDA-MB-231
m: HeLa

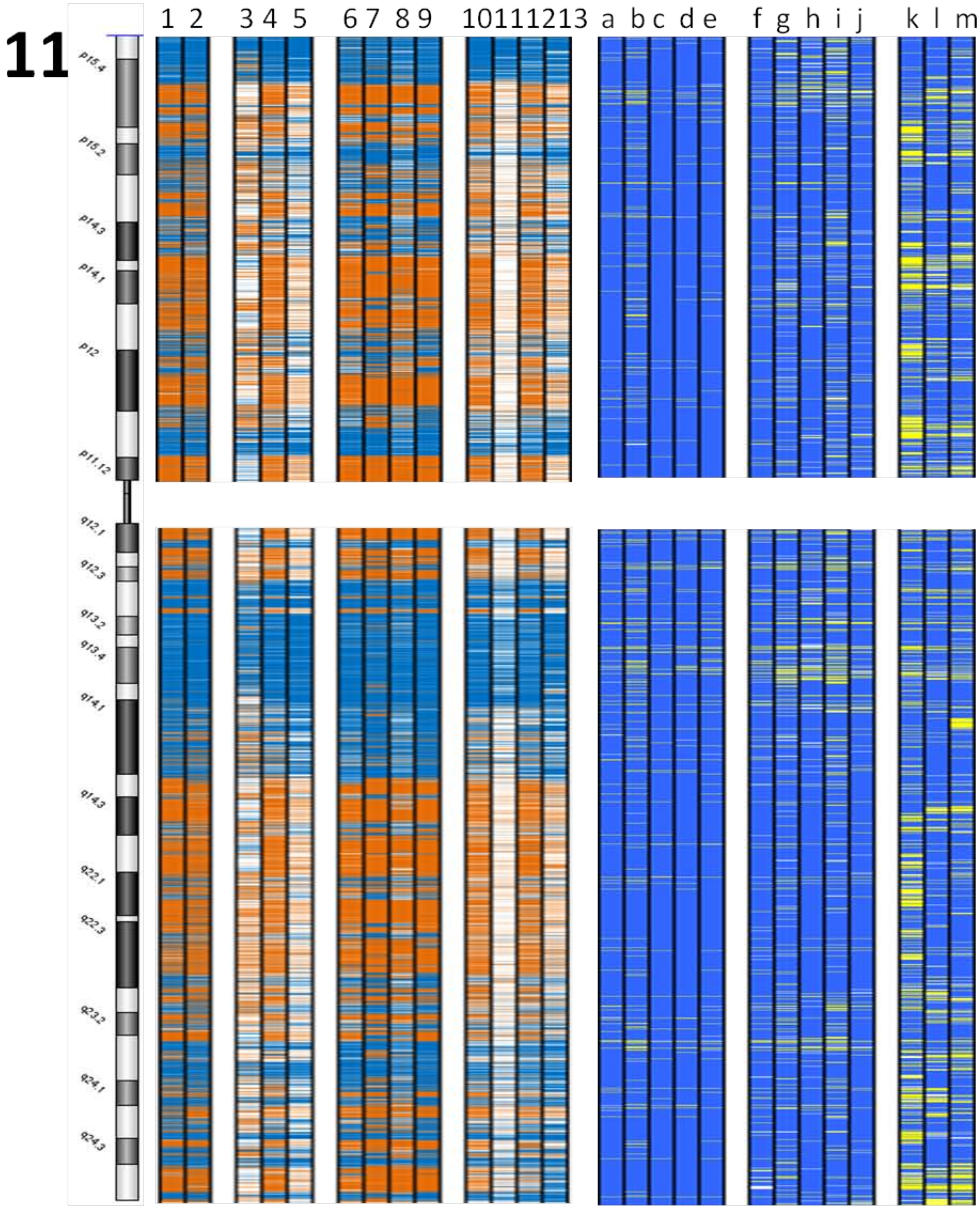

McrBC method

1: FaDu HIF
2: Fadu
3: Malhavu
4: CL15
5: MDA-MB-231
6: MCF7
7: HepG2
8: huh7
9: CL10
10: breast tumor tissue
11: brain tumor tissue(no.RT619)
12: brain tumor tissue(no.t891)
13: brain tumor tissue(no.RT297)

TspRI-ExoIII method

a: brain normal tissue
b: leukocyte normal tissue
c: testis normal tissue
d: liver normal tissue
e: breast normal tissue
f: breast tumor tissue (no. 146)
g: breast tumor tissue (no. 125)
h: breast tumor tissue (no. 93)
i: breast tumor tissue (no. 89)
j: brain tumor tissue (no. SIR)
k: MCF7
l: MDA-MB-231
m: HeLa

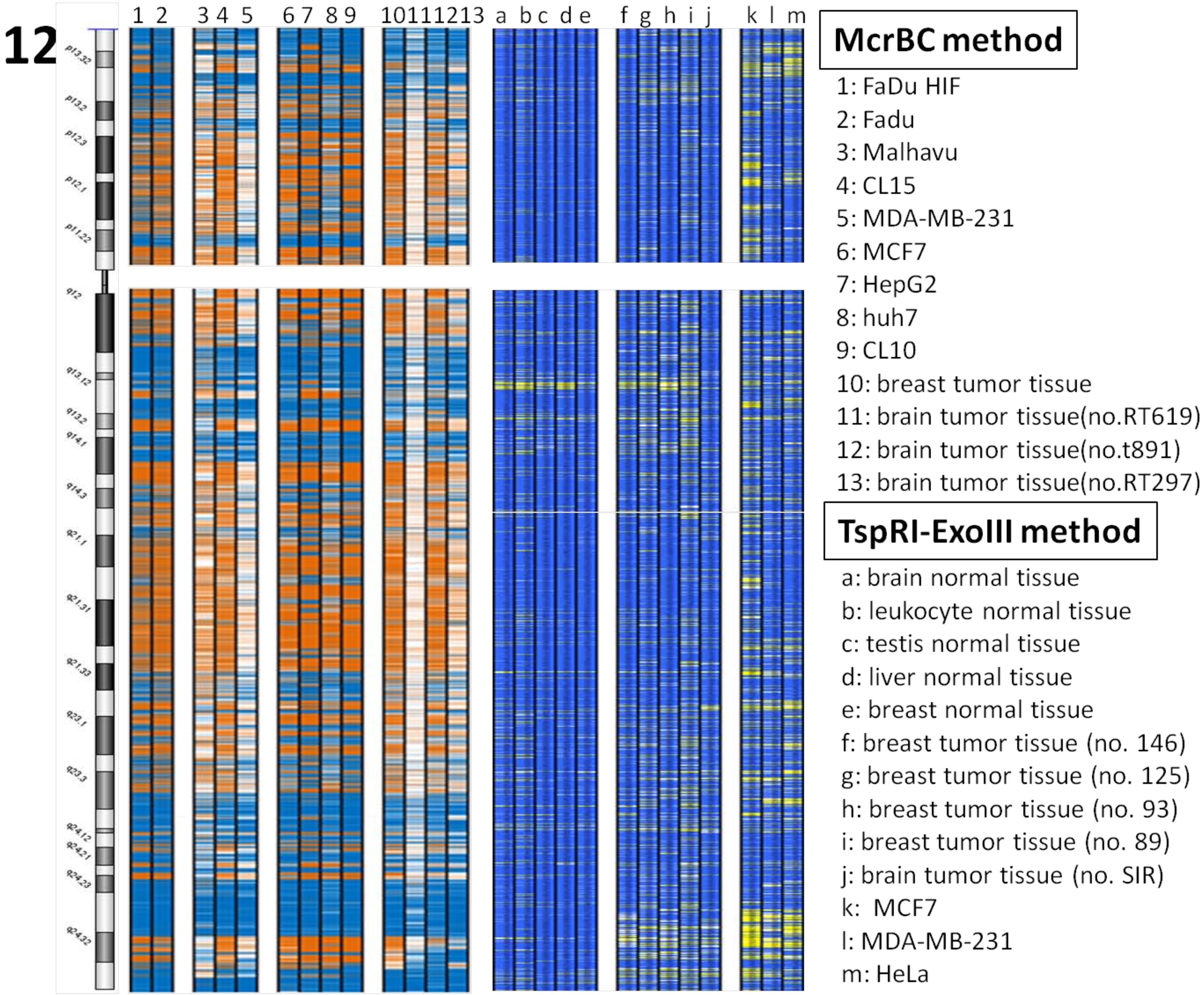

12
1 2 3 4 5 6 7 8 9 10 11 12 13 a b c d e f g h i j k l m
McrBC method
1: FaDu HIF
2: Fadu
3: Malhavu
4: CL15
5: MDA-MB-231
6: MCF7
7: HepG2
8: huh7
9: CL10
10: breast tumor tissue
11: brain tumor tissue(no.RT619)
12: brain tumor tissue(no.t891)
13: brain tumor tissue(no.RT297)
TspRI-ExoIII method
a: brain normal tissue
b: leukocyte normal tissue
c: testis normal tissue
d: liver normal tissue
e: breast normal tissue
f: breast tumor tissue (no. 146)
g: breast tumor tissue (no. 125)
h: breast tumor tissue (no. 93)
i: breast tumor tissue (no. 89)
j: brain tumor tissue (no. SIR)
k: MCF7
l: MDA-MB-231
m: HeLa

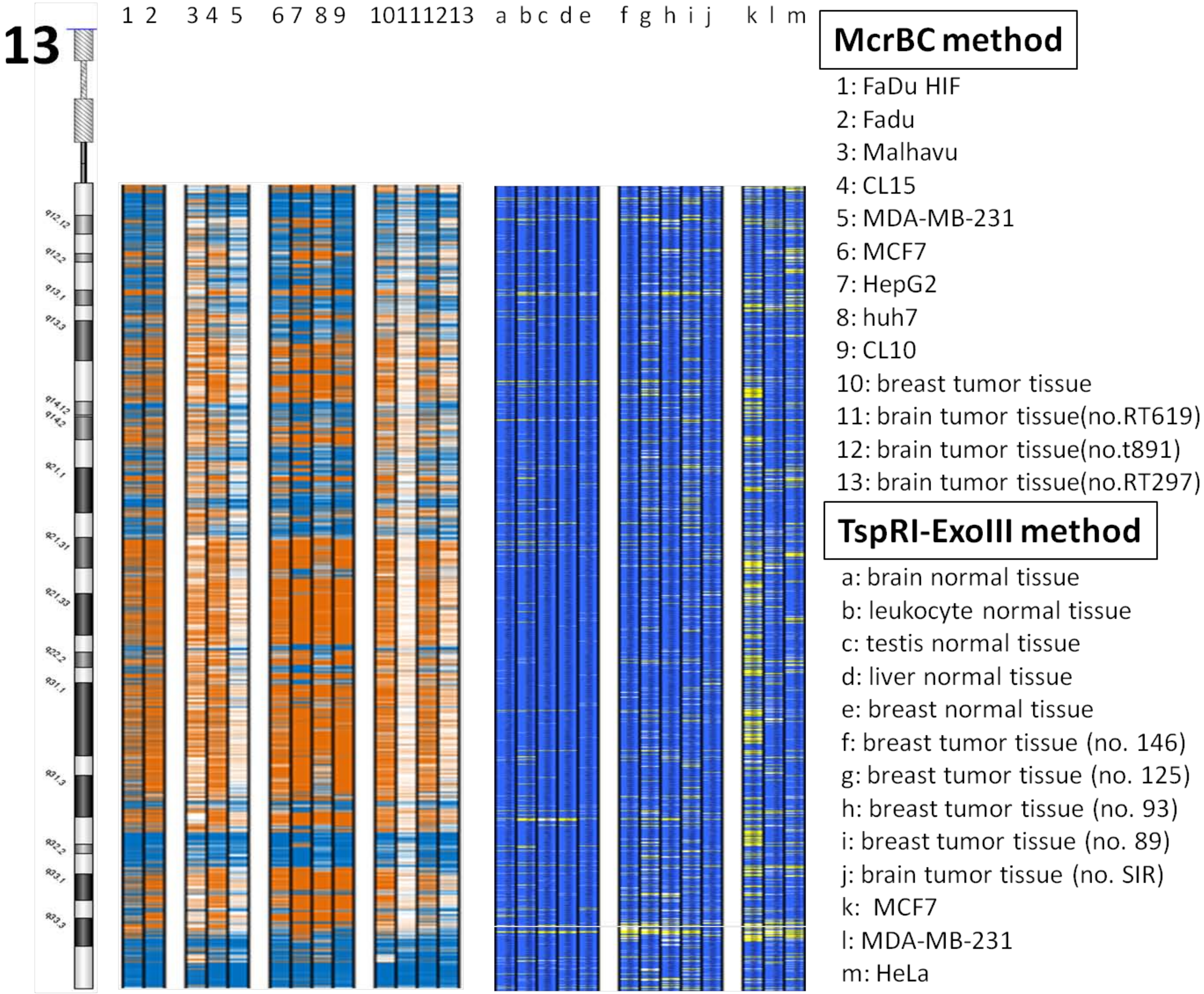
13
1 2 3 4 5 6 7 8 9 10111213
a b c d e f g h i j k l m
McrBC method
1: FaDu HIF
2: Fadu
3: Malhavu
4: CL15
5: MDA-MB-231
6: MCF7
7: HepG2
8: huh7
9: CL10
10: breast tumor tissue
11: brain tumor tissue(no.RT619)
12: brain tumor tissue(no.t891)
13: brain tumor tissue(no.RT297)
TspRI-ExoIII method
a: brain normal tissue
b: leukocyte normal tissue
c: testis normal tissue
d: liver normal tissue
e: breast normal tissue
f: breast tumor tissue (no. 146)
g: breast tumor tissue (no. 125)
h: breast tumor tissue (no. 93)
i: breast tumor tissue (no. 89)
j: brain tumor tissue (no. SIR)
k: MCF7
l: MDA-MB-231
m: HeLa

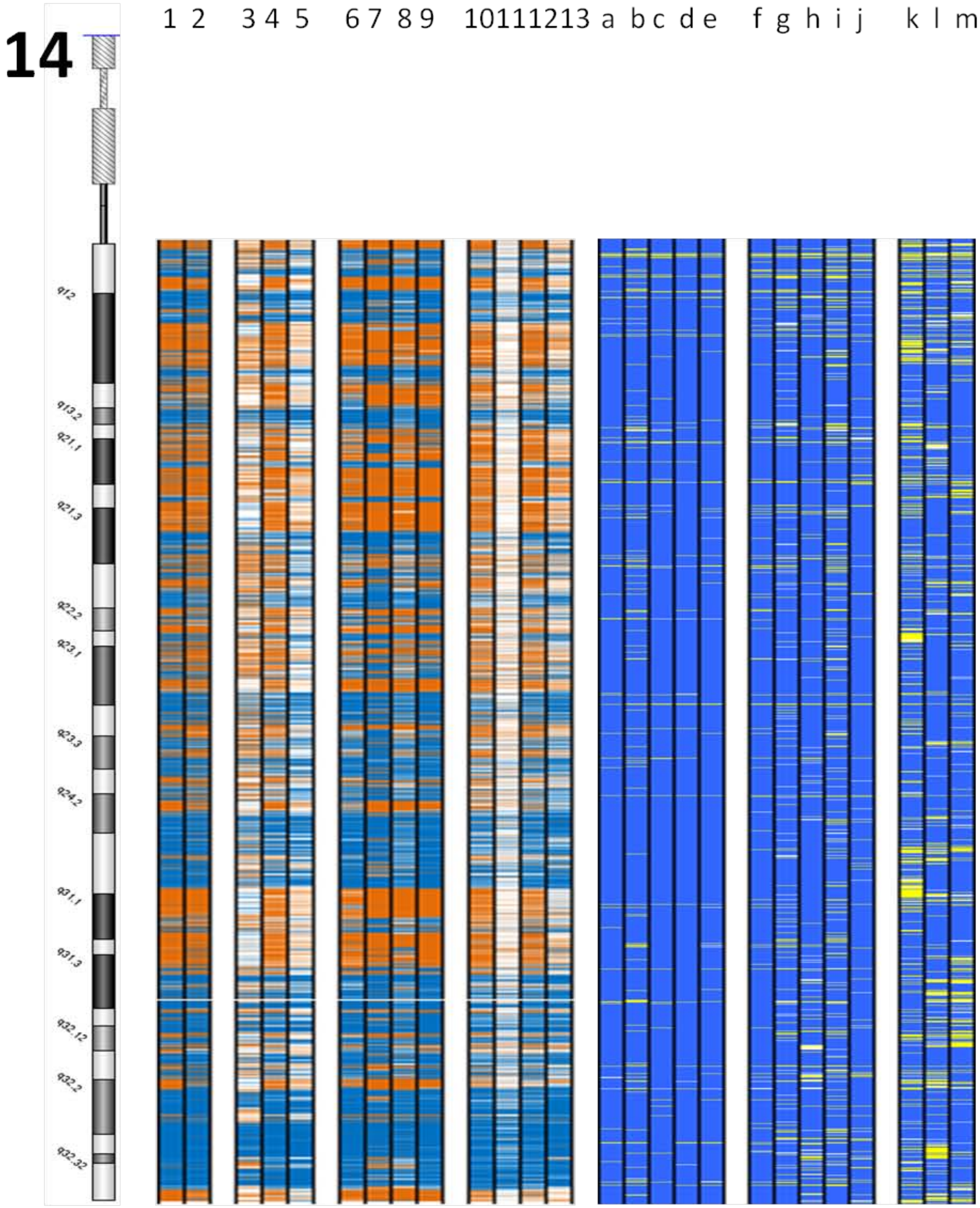

McrBC method

1: FaDu HIF
2: Fadu
3: Malhavu
4: CL15
5: MDA-MB-231
6: MCF7
7: HepG2
8: huh7
9: CL10
10: breast tumor tissue
11: brain tumor tissue(no.RT619)
12: brain tumor tissue(no.t891)
13: brain tumor tissue(no.RT297)

TspRI-ExoIII method

a: brain normal tissue
b: leukocyte normal tissue
c: testis normal tissue
d: liver normal tissue
e: breast normal tissue
f: breast tumor tissue (no. 146)
g: breast tumor tissue (no. 125)
h: breast tumor tissue (no. 93)
i: breast tumor tissue (no. 89)
j: brain tumor tissue (no. SIR)
k: MCF7
l: MDA-MB-231
m: HeLa

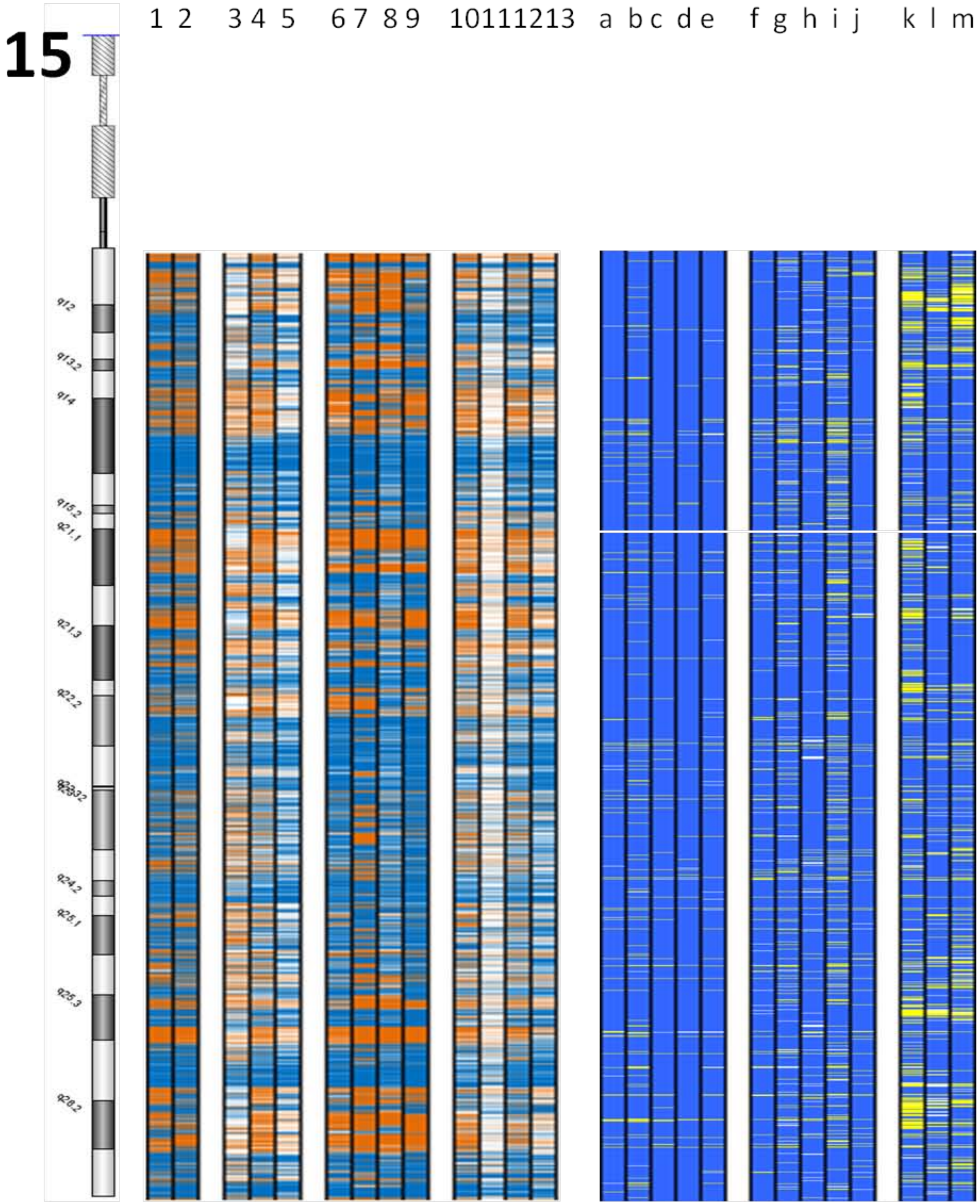

McrBC method

1: FaDu HIF
2: Fadu
3: Malhavu
4: CL15
5: MDA-MB-231
6: MCF7
7: HepG2
8: huh7
9: CL10
10: breast tumor tissue
11: brain tumor tissue(no.RT619)
12: brain tumor tissue(no.t891)
13: brain tumor tissue(no.RT297)

TspRI-ExoIII method

a: brain normal tissue
b: leukocyte normal tissue
c: testis normal tissue
d: liver normal tissue
e: breast normal tissue
f: breast tumor tissue (no. 146)
g: breast tumor tissue (no. 125)
h: breast tumor tissue (no. 93)
i: breast tumor tissue (no. 89)
j: brain tumor tissue (no. SIR)
k: MCF7
l: MDA-MB-231
m: HeLa

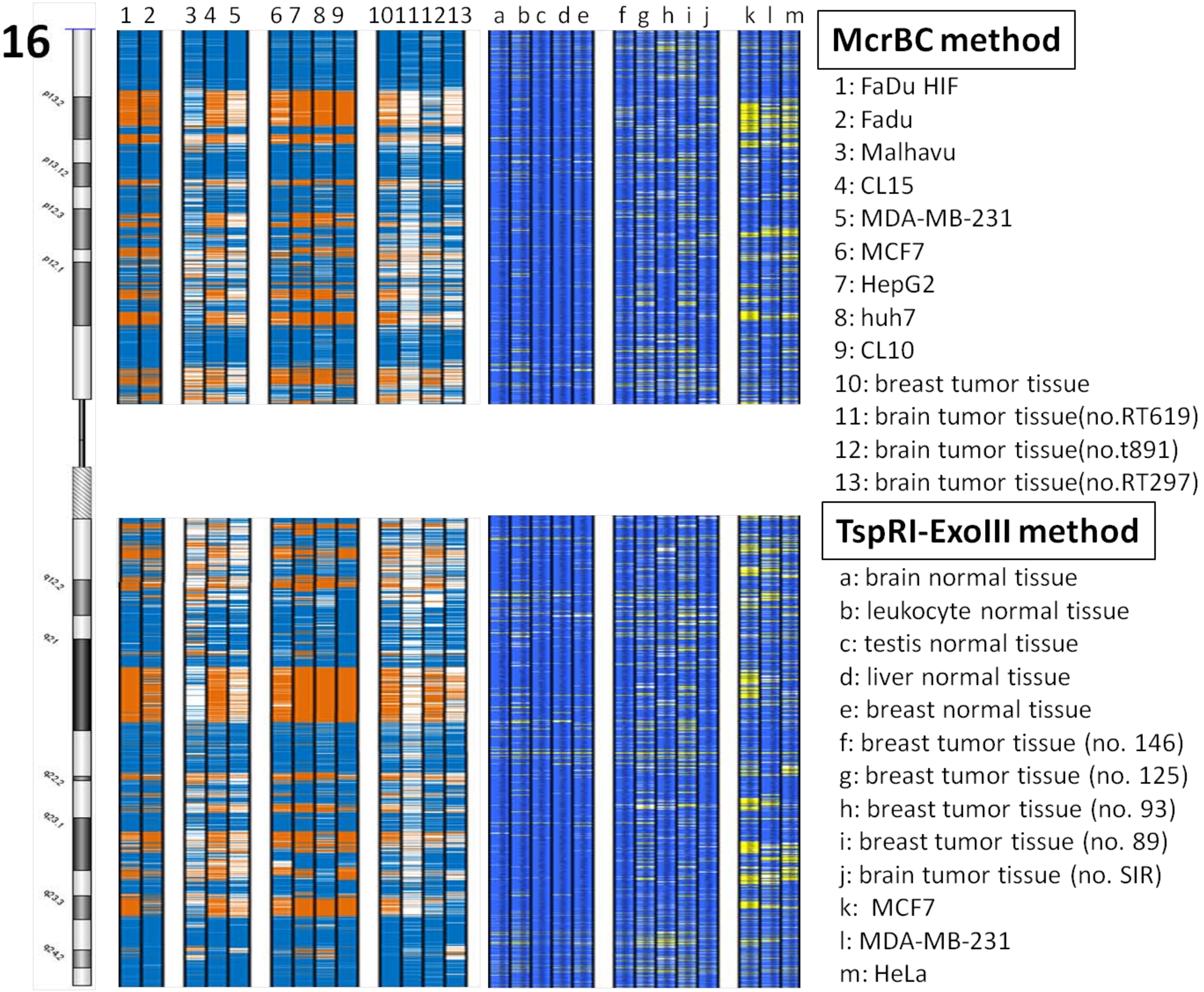
16
1 2 3 4 5 6 7 8 9 10 11 12 13 a b c d e f g h i j k l m
McrBC method
1: FaDu HIF
2: Fadu
3: Malhavu
4: CL15
5: MDA-MB-231
6: MCF7
7: HepG2
8: huh7
9: CL10
10: breast tumor tissue
11: brain tumor tissue(no.RT619)
12: brain tumor tissue(no.t891)
13: brain tumor tissue(no.RT297)
TspRI-ExoIII method
a: brain normal tissue
b: leukocyte normal tissue
c: testis normal tissue
d: liver normal tissue
e: breast normal tissue
f: breast tumor tissue (no. 146)
g: breast tumor tissue (no. 125)
h: breast tumor tissue (no. 93)
i: breast tumor tissue (no. 89)
j: brain tumor tissue (no. SIR)
k: MCF7
l: MDA-MB-231
m: HeLa

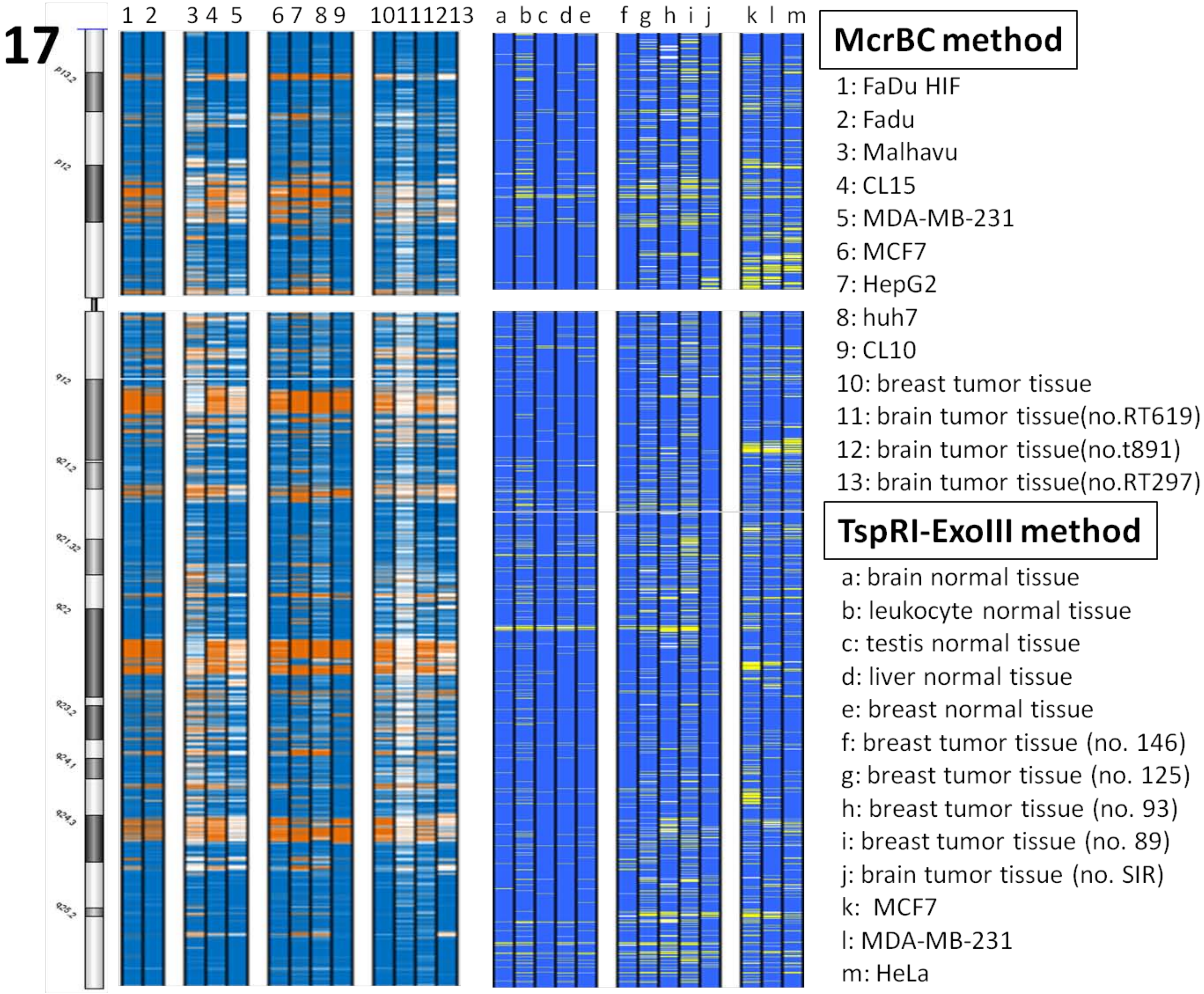
17
1 2 3 4 5 6 7 8 9 10 11 12 13
a b c d e f g h i j k l m
p13.2
p12
q12
q21.2
q21.32
q22
q23.2
q24.1
q24.3
q25.2
McrBC method
1: FaDu HIF
2: Fadu
3: Malhavu
4: CL15
5: MDA-MB-231
6: MCF7
7: HepG2
8: huh7
9: CL10
10: breast tumor tissue
11: brain tumor tissue(no.RT619)
12: brain tumor tissue(no.t891)
13: brain tumor tissue(no.RT297)
TspRI-ExoIII method
a: brain normal tissue
b: leukocyte normal tissue
c: testis normal tissue
d: liver normal tissue
e: breast normal tissue
f: breast tumor tissue (no. 146)
g: breast tumor tissue (no. 125)
h: breast tumor tissue (no. 93)
i: breast tumor tissue (no. 89)
j: brain tumor tissue (no. SIR)
k: MCF7
l: MDA-MB-231
m: HeLa

18

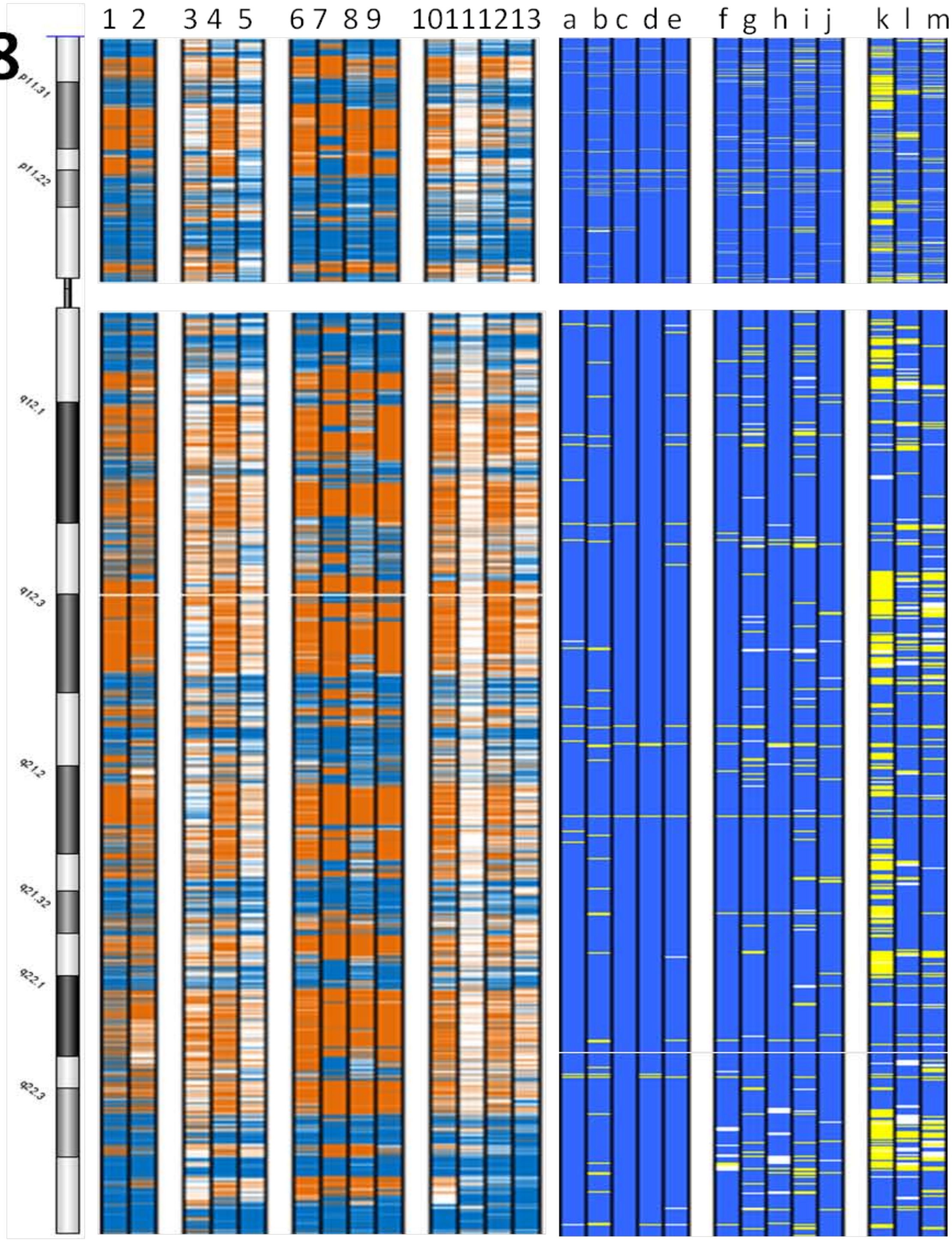

McrBC method

1: FaDu HIF
2: Fadu
3: Malhavu
4: CL15
5: MDA-MB-231
6: MCF7
7: HepG2
8: huh7
9: CL10
10: breast tumor tissue
11: brain tumor tissue(no.RT619)
12: brain tumor tissue(no.t891)
13: brain tumor tissue(no.RT297)

TspRI-ExoIII method

a: brain normal tissue
b: leukocyte normal tissue
c: testis normal tissue
d: liver normal tissue
e: breast normal tissue
f: breast tumor tissue (no. 146)
g: breast tumor tissue (no. 125)
h: breast tumor tissue (no. 93)
i: breast tumor tissue (no. 89)
j: brain tumor tissue (no. SIR)
k: MCF7
l: MDA-MB-231
m: HeLa

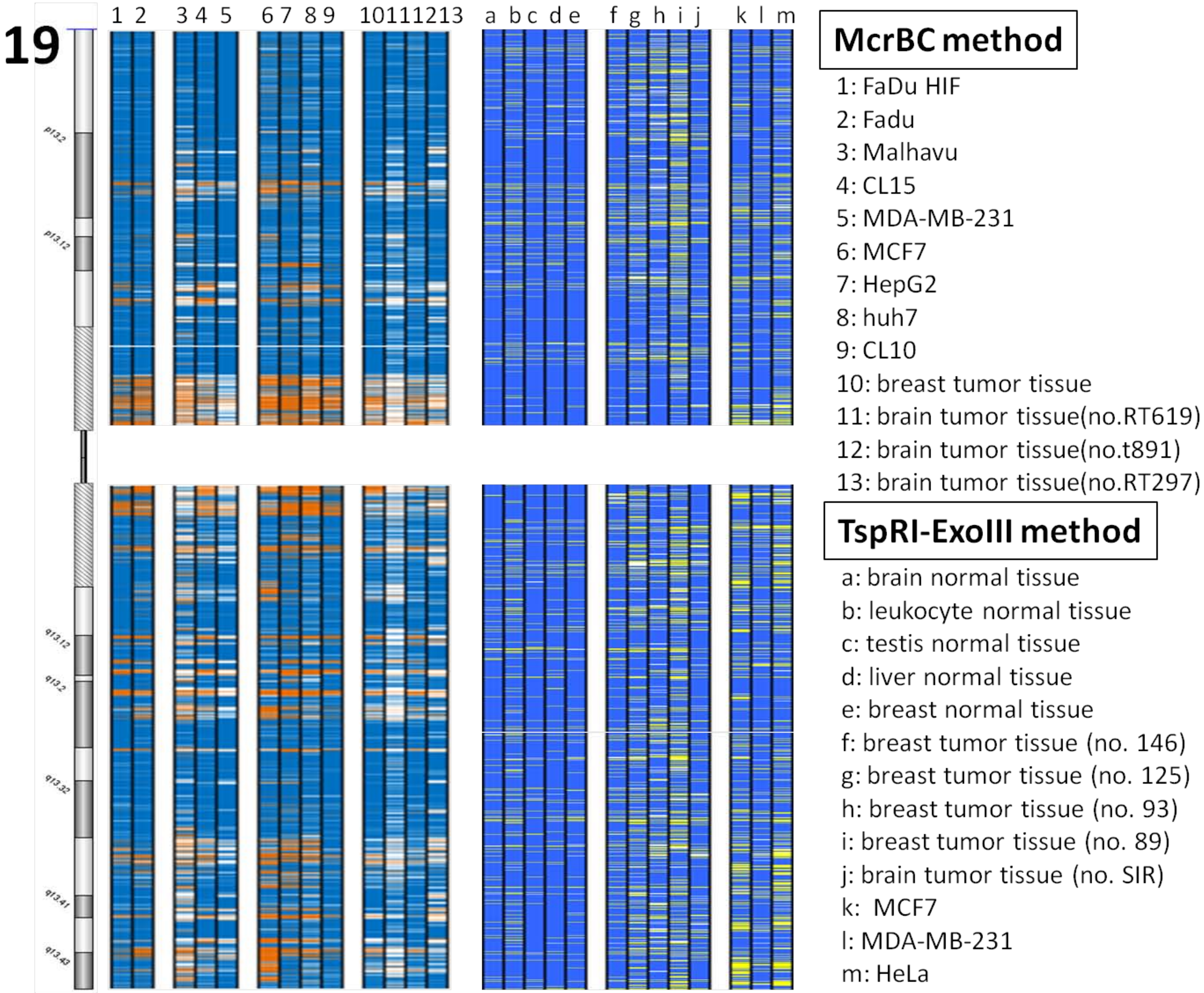
19
1 2 3 4 5 6 7 8 9 10 11 12 13
a b c d e f g h i j k l m
McrBC method
1: FaDu HIF
2: Fadu
3: Malhavu
4: CL15
5: MDA-MB-231
6: MCF7
7: HepG2
8: huh7
9: CL10
10: breast tumor tissue
11: brain tumor tissue(no.RT619)
12: brain tumor tissue(no.t891)
13: brain tumor tissue(no.RT297)
TspRI-ExoIII method
a: brain normal tissue
b: leukocyte normal tissue
c: testis normal tissue
d: liver normal tissue
e: breast normal tissue
f: breast tumor tissue (no. 146)
g: breast tumor tissue (no. 125)
h: breast tumor tissue (no. 93)
i: breast tumor tissue (no. 89)
j: brain tumor tissue (no. SIR)
k: MCF7
l: MDA-MB-231
m: HeLa

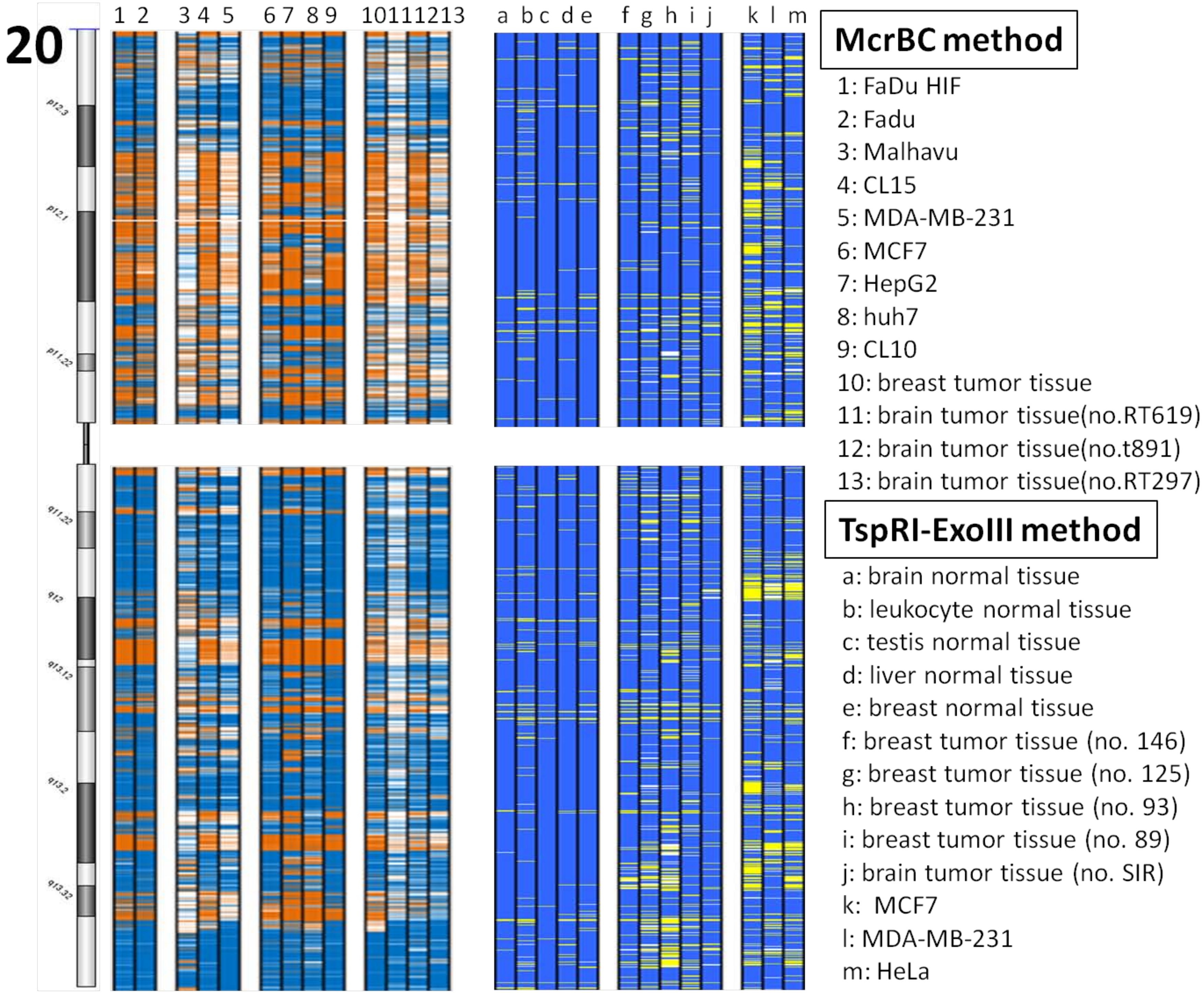
20
1 2 3 4 5 6 7 8 9 10 11 12 13
a b c d e f g h i j k l m
McrBC method
1: FaDu HIF
2: Fadu
3: Malhavu
4: CL15
5: MDA-MB-231
6: MCF7
7: HepG2
8: huh7
9: CL10
10: breast tumor tissue
11: brain tumor tissue(no.RT619)
12: brain tumor tissue(no.t891)
13: brain tumor tissue(no.RT297)
TspRI-ExoIII method
a: brain normal tissue
b: leukocyte normal tissue
c: testis normal tissue
d: liver normal tissue
e: breast normal tissue
f: breast tumor tissue (no. 146)
g: breast tumor tissue (no. 125)
h: breast tumor tissue (no. 93)
i: breast tumor tissue (no. 89)
j: brain tumor tissue (no. SIR)
k: MCF7
l: MDA-MB-231
m: HeLa

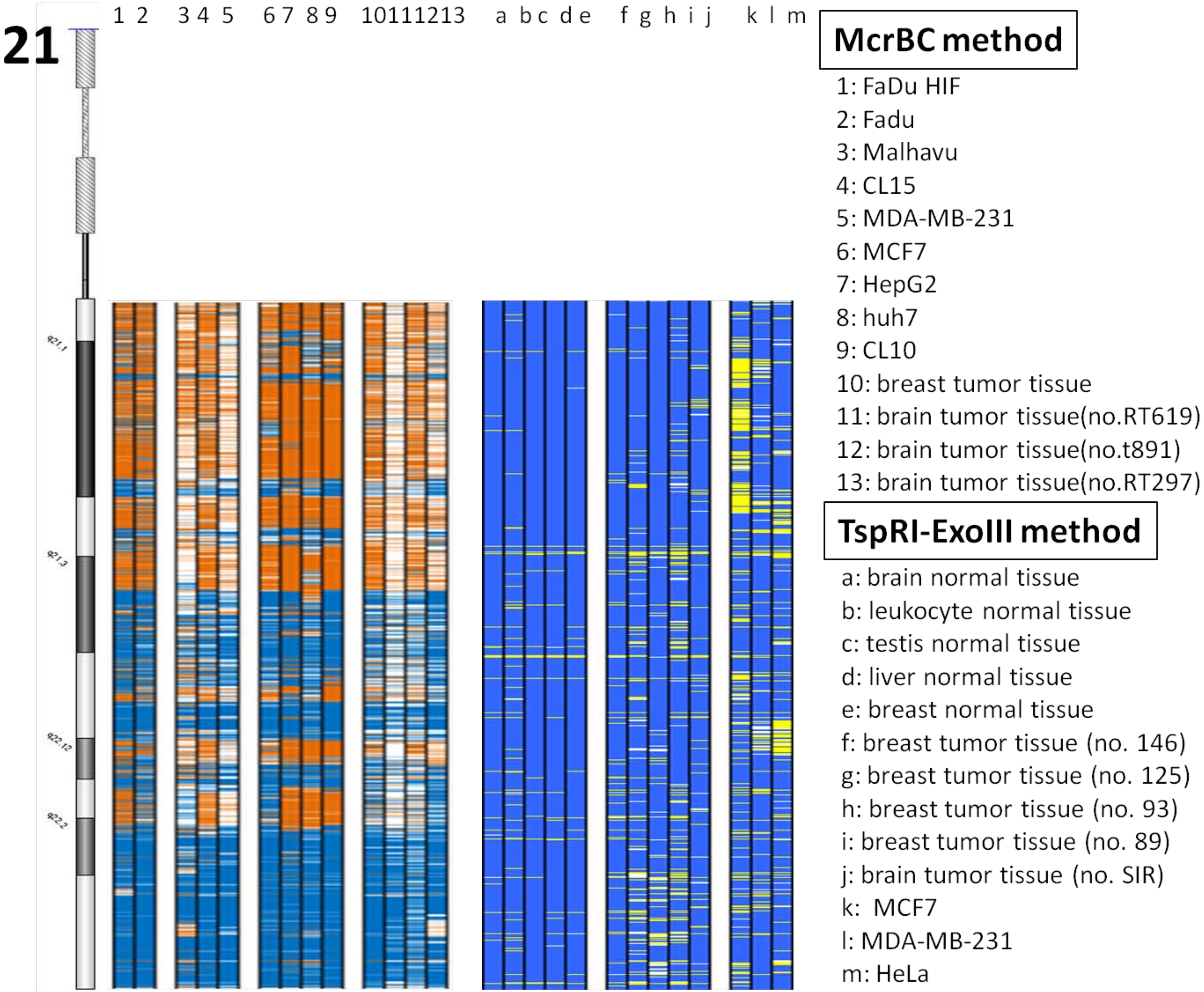
21
1 2 3 4 5 6 7 8 9 10 11 12 13 a b c d e f g h i j k l m
q21.1
q21.3
q22.12
q22.2
McrBC method
1: FaDu HIF
2: Fadu
3: Malhavu
4: CL15
5: MDA-MB-231
6: MCF7
7: HepG2
8: huh7
9: CL10
10: breast tumor tissue
11: brain tumor tissue(no.RT619)
12: brain tumor tissue(no.t891)
13: brain tumor tissue(no.RT297)
TspRI-ExoIII method
a: brain normal tissue
b: leukocyte normal tissue
c: testis normal tissue
d: liver normal tissue
e: breast normal tissue
f: breast tumor tissue (no. 146)
g: breast tumor tissue (no. 125)
h: breast tumor tissue (no. 93)
i: breast tumor tissue (no. 89)
j: brain tumor tissue (no. SIR)
k: MCF7
l: MDA-MB-231
m: HeLa

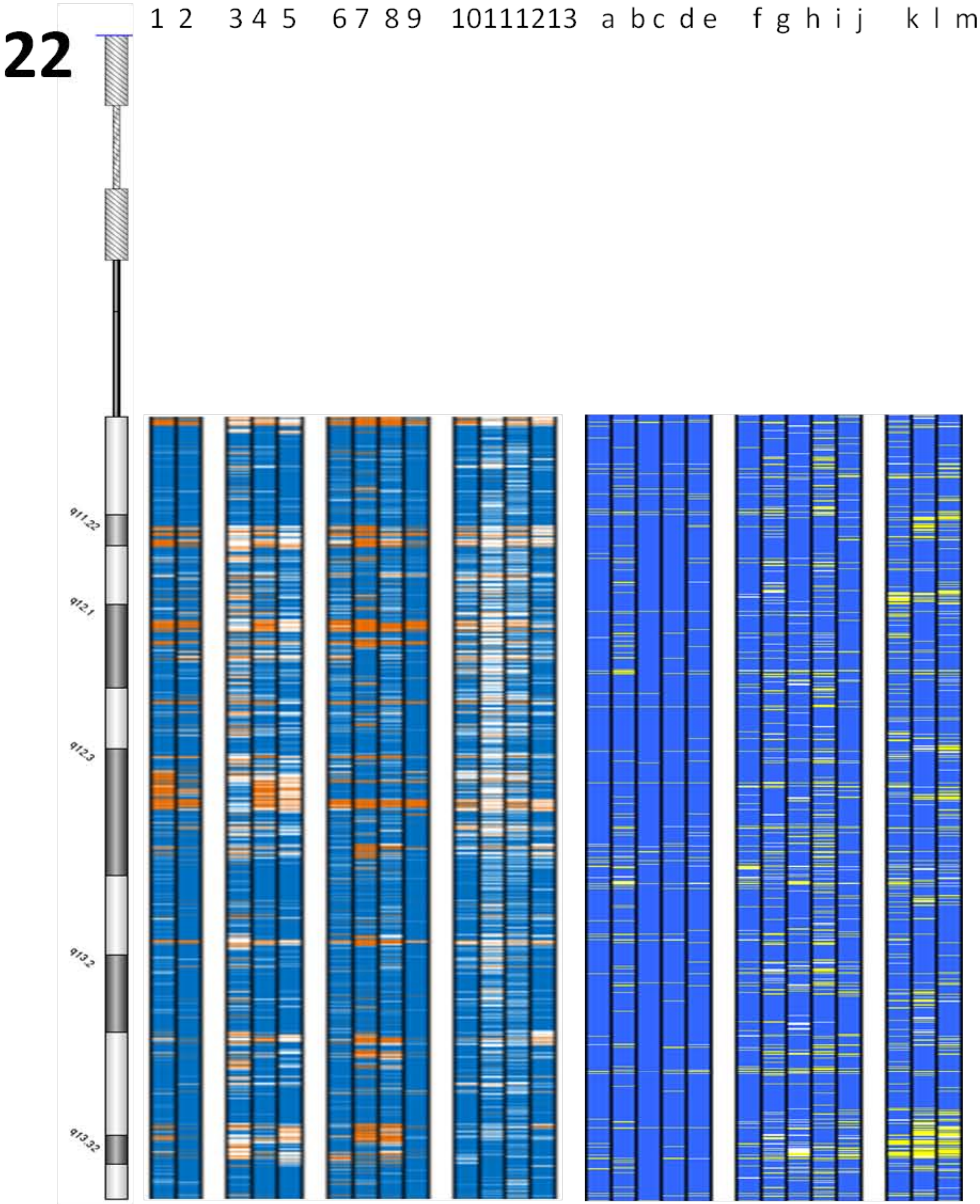

McrBC method

1: FaDu HIF
2: Fadu
3: Malhavu
4: CL15
5: MDA-MB-231
6: MCF7
7: HepG2
8: huh7
9: CL10
10: breast tumor tissue
11: brain tumor tissue(no.RT619)
12: brain tumor tissue(no.t891)
13: brain tumor tissue(no.RT297)

TspRI-ExoIII method

a: brain normal tissue
b: leukocyte normal tissue
c: testis normal tissue
d: liver normal tissue
e: breast normal tissue
f: breast tumor tissue (no. 146)
g: breast tumor tissue (no. 125)
h: breast tumor tissue (no. 93)
i: breast tumor tissue (no. 89)
j: brain tumor tissue (no. SIR)
k: MCF7
l: MDA-MB-231
m: HeLa

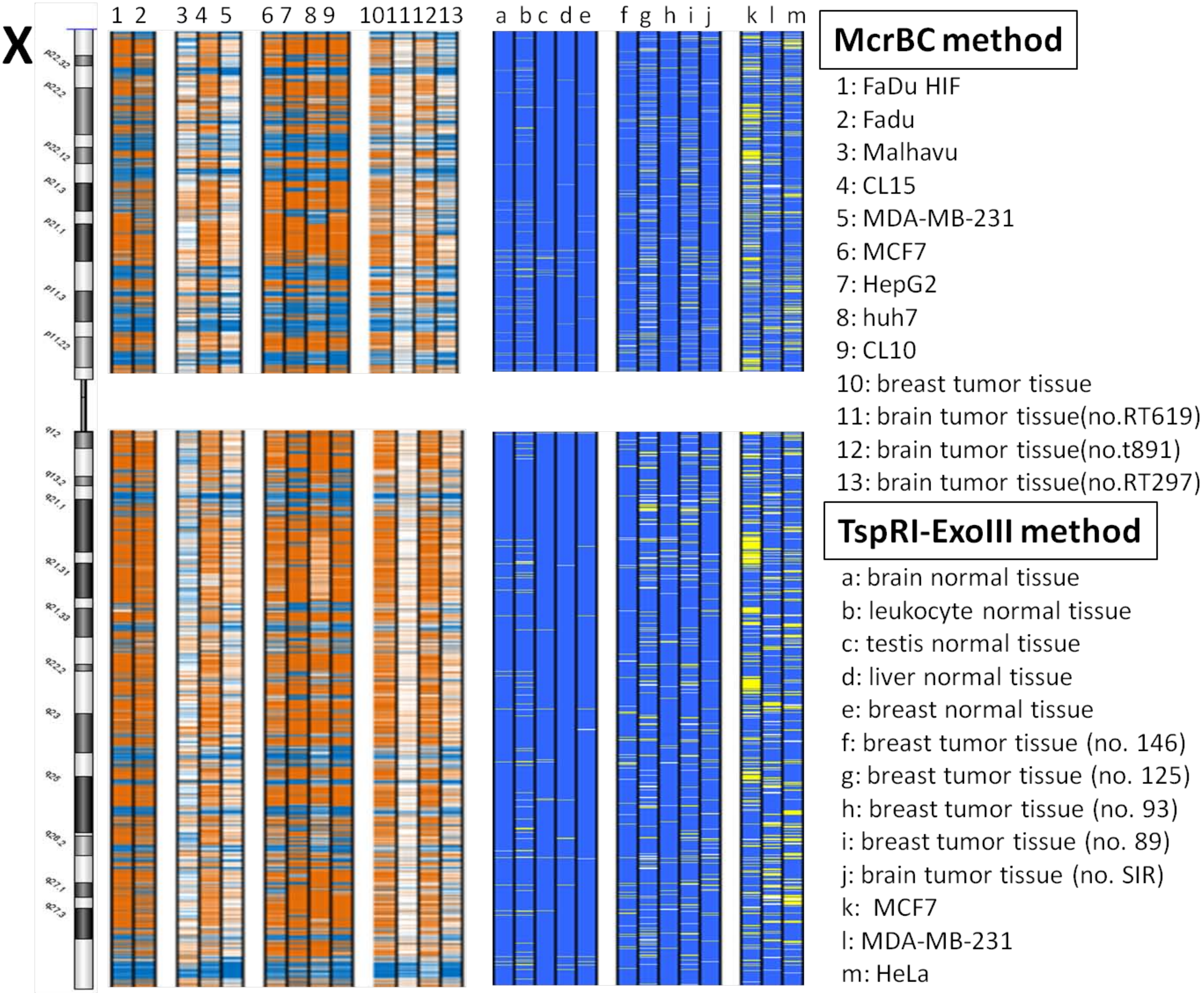
X
1 2 3 4 5 6 7 8 9 10 11 12 13
a b c d e f g h i j k l m
McrBC method
1: FaDu HIF
2: Fadu
3: Malhavu
4: CL15
5: MDA-MB-231
6: MCF7
7: HepG2
8: huh7
9: CL10
10: breast tumor tissue
11: brain tumor tissue(no.RT619)
12: brain tumor tissue(no.t891)
13: brain tumor tissue(no.RT297)
TspRI-ExoIII method
a: brain normal tissue
b: leukocyte normal tissue
c: testis normal tissue
d: liver normal tissue
e: breast normal tissue
f: breast tumor tissue (no. 146)
g: breast tumor tissue (no. 125)
h: breast tumor tissue (no. 93)
i: breast tumor tissue (no. 89)
j: brain tumor tissue (no. SIR)
k: MCF7
l: MDA-MB-231
m: HeLa

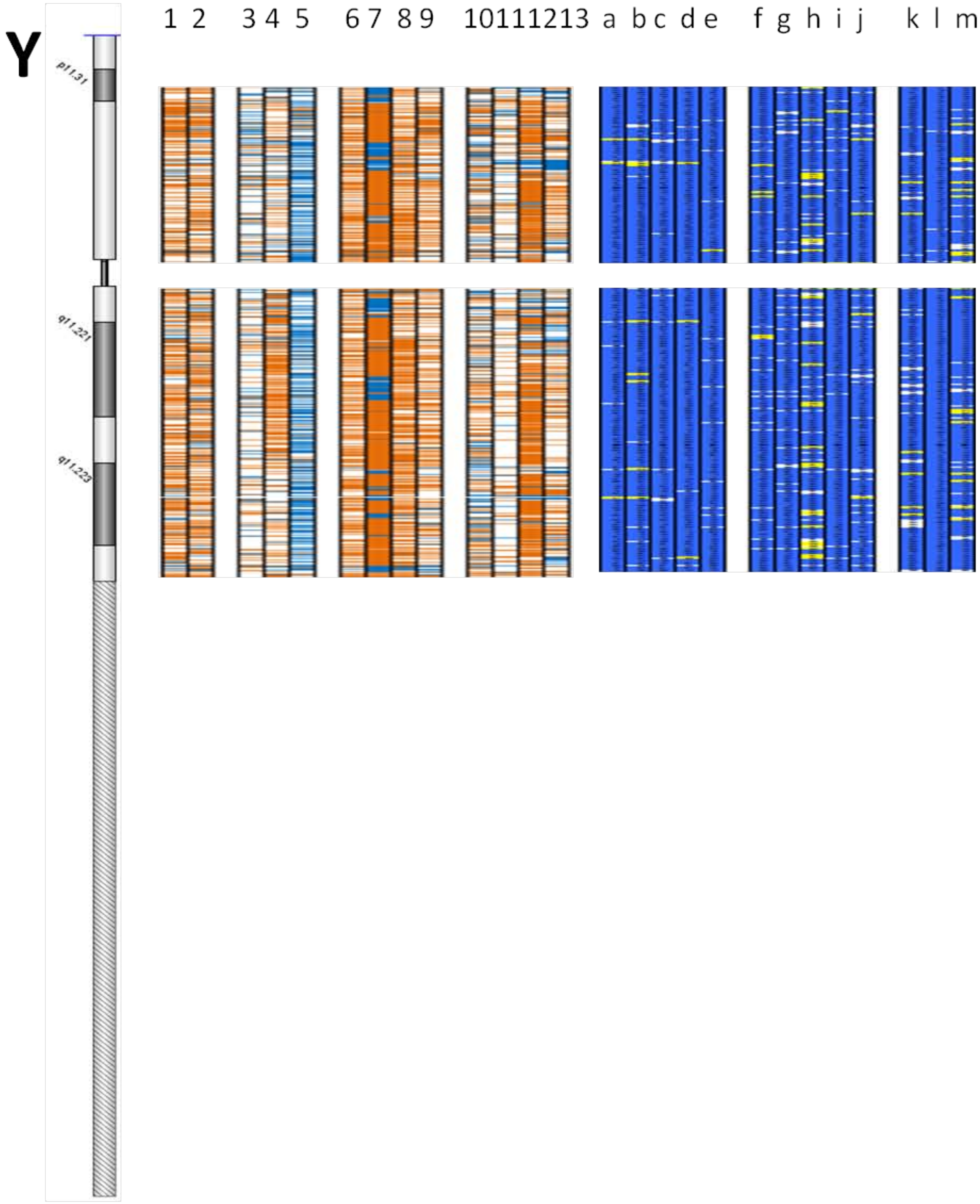

McrBC method

1: FaDu HIF
2: Fadu
3: Malhavu
4: CL15
5: MDA-MB-231
6: MCF7
7: HepG2
8: huh7
9: CL10
10: breast tumor tissue
11: brain tumor tissue(no.RT619)
12: brain tumor tissue(no.t891)
13: brain tumor tissue(no.RT297)

TspRI-ExoIII method

a: brain normal tissue
b: leukocyte normal tissue
c: testis normal tissue
d: liver normal tissue
e: breast normal tissue
f: breast tumor tissue (no. 146)
g: breast tumor tissue (no. 125)
h: breast tumor tissue (no. 93)
i: breast tumor tissue (no. 89)
j: brain tumor tissue (no. SIR)
k: MCF7
l: MDA-MB-231
m: HeLa

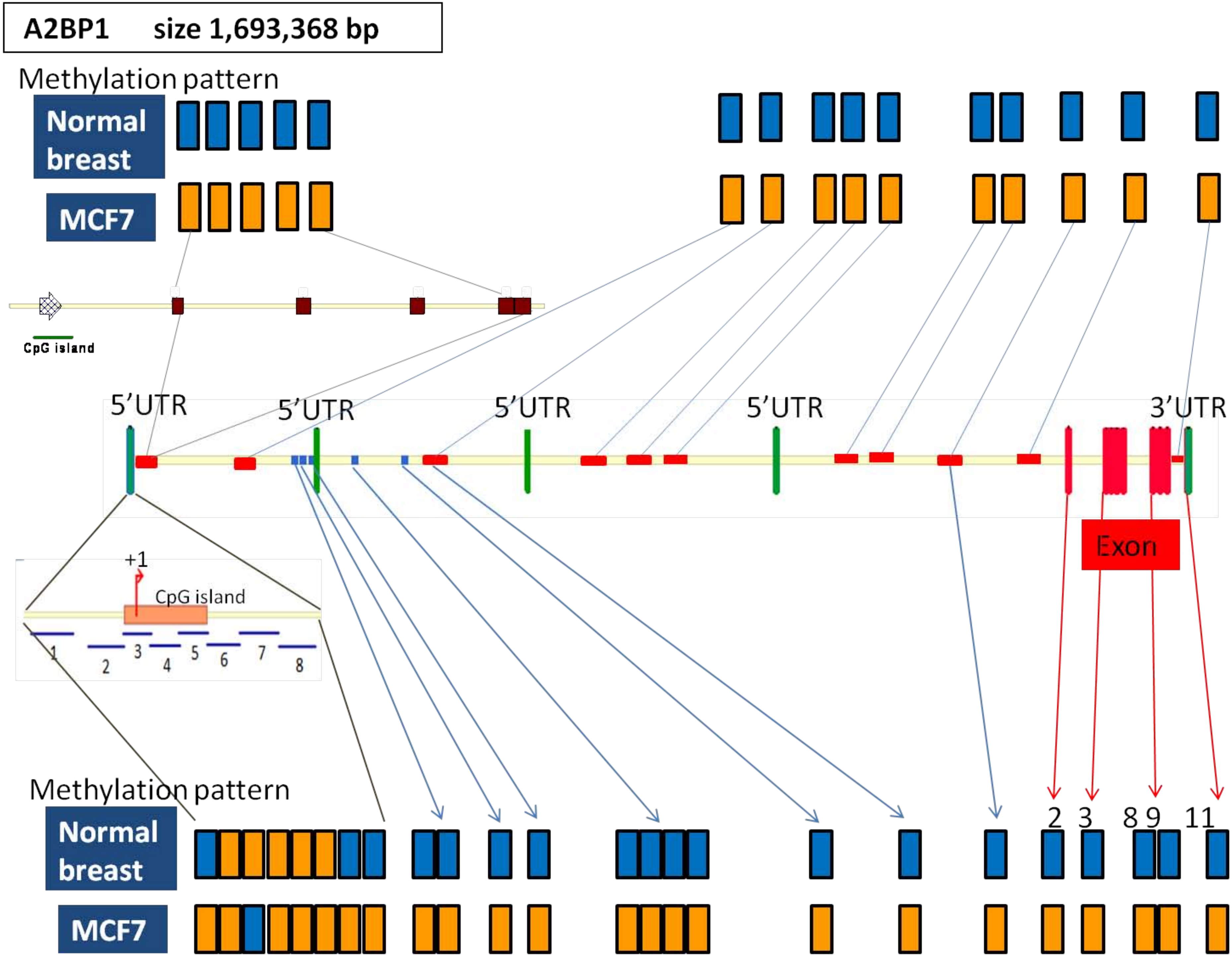
A2BP1 size 1,693,368 bp
Methylation pattern
Normal breast
MCF7
CpG island
5'UTR
5'UTR
5'UTR
5'UTR
3'UTR
Exon
+1
CpG island
1
2
3
4
5
6
7
8
Methylation pattern
Normal breast
MCF7
2
3
8
9
11

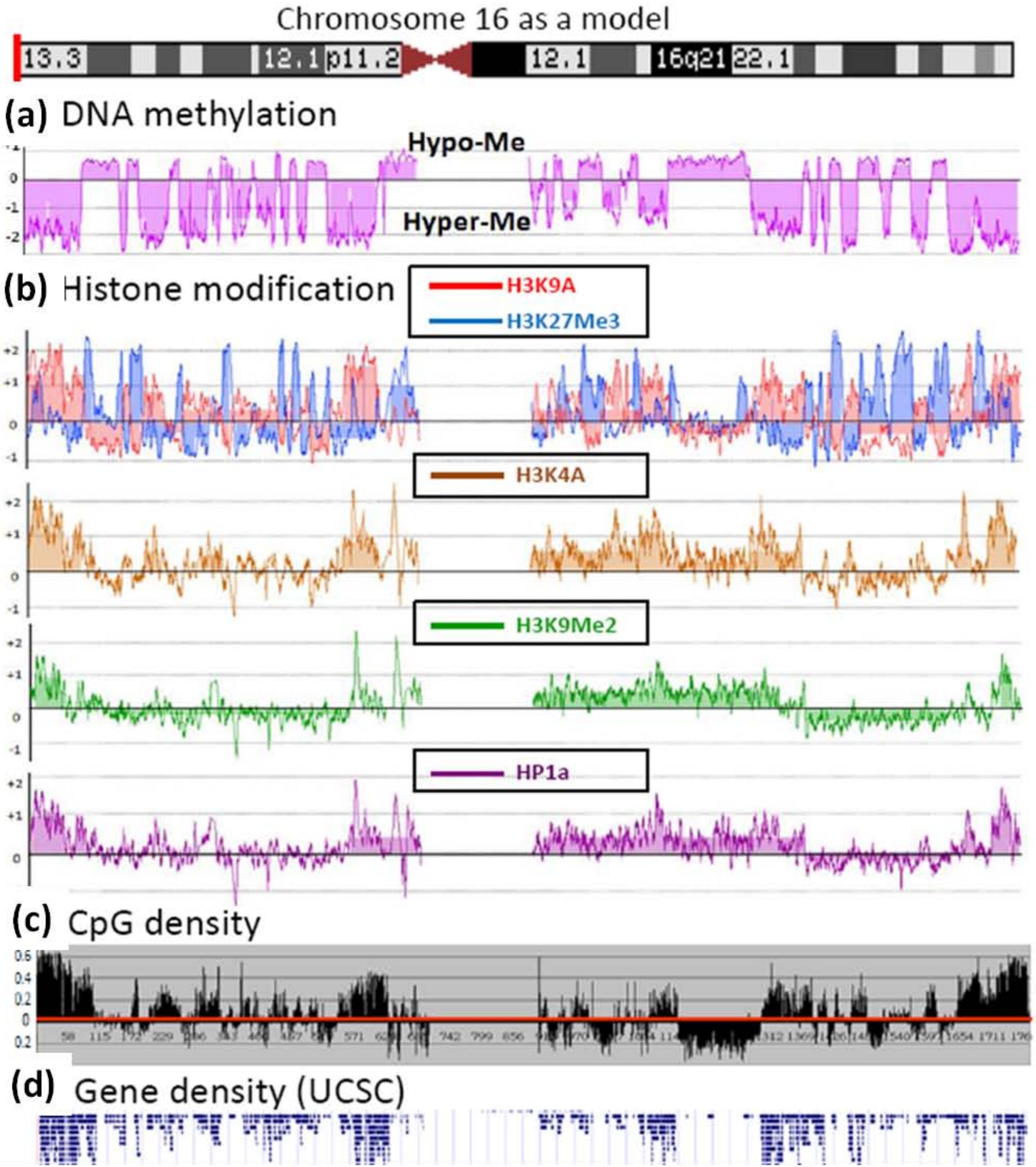

Chromosome 16 as a model
13.3
12.1
p11.2
12.1
16q21
22.1
(a) DNA methylation
Hypo-Me
Hyper-Me
(b) Histone modification
H3K9A
H3K27Me3
H3K4A
H3K9Me2
HP1a
(c) CpG density
(d) Gene density (UCSC)
